# Artifact geochemistry demonstrates long-distance voyaging in the Polynesian Outliers

**DOI:** 10.1126/sciadv.adf4487

**Published:** 2023-04-21

**Authors:** Aymeric Hermann, Pamela Gutiérrez, Catherine Chauvel, René Maury, Céline Liorzou, Edson Willie, Iarawai Phillip, Robert Forkel, Christoph Rzymski, Stuart Bedford

**Affiliations:** ^1^ UMR 8068 Temps, CNRS, F-92023 Nanterre, France.; ^2^ Department of Linguistic and Cultural Evolution, MPI-EVA, D-04103 Leipzig, Germany.; ^3^ Université Paris Cité, Institut de Physique du Globe de Paris, CNRS, F-75005 Paris, France.; ^4^ Université de Brest, UMR6538 Géosciences Océan, Institut Universitaire Européen de la Mer, CNRS, F-29280 Plouzané, France.; ^5^ Vanuatu National Museum, Vanuatu Cultural Centre, P.O. Box 184, Port-Vila, Vanuatu.; ^6^ Department of Archaeology and Natural History, College of Asia and the Pacific, The Australian National University, Canberra, ACT 2601, Australia.

## Abstract

Although the peopling of Remote Oceania is well-documented as a general process of eastward migrations from Island Southeast Asia and Near Oceania toward the archipelagos of Remote Oceania, the origin and the development of Polynesian societies in the Western Pacific (Polynesian Outliers), far away from the Polynesian triangle, remain unclear. Here, we present a large-scale geochemical sourcing study of stone artifacts excavated from archeological sites in central Vanuatu, the Solomon Islands, and the Caroline Islands and provide unambiguous evidence of multiple long-distance voyages, with exotic stone materials being transported up to 2500 kilometers from their source. Our results emphasize high mobility in the Western Pacific during the last millennium CE and offer insights on the scale and timing of contacts between the Polynesian Outliers, their neighbors in the Western Pacific, and societies of Western Polynesia.

## Teaser

Geochemical fingerprinting of stone artefacts reveals long-distance voyaging among Pacific Islands during the last millennium.

## INTRODUCTION

**Fig. 1.**
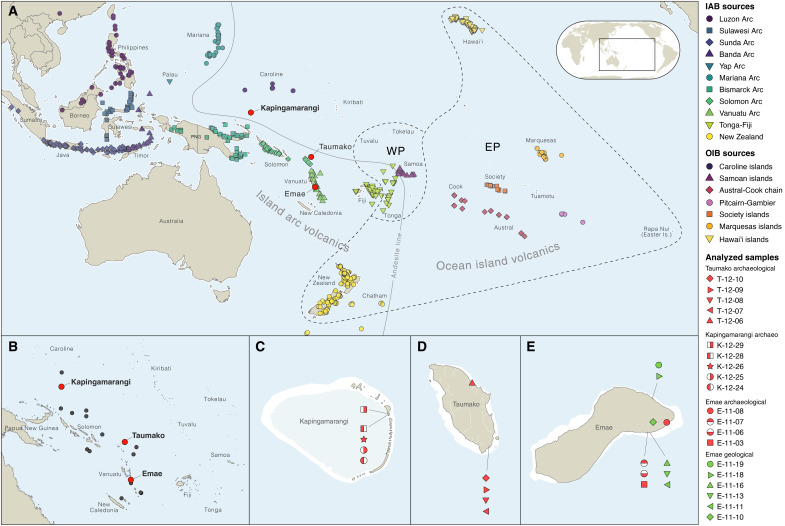
Pacific islands and provenance of analyzed samples. (**A**) Location of analyzed samples and potential sources among island arc (IAB) and ocean island (OIB) domains west and east of the Andesite line (*35*). (**B**) Location of the Polynesian outliers, outside of western Polynesia (WP) and eastern Polynesia (EP). (**C**) Archeological provenance of analyzed samples in Kapingamarangi atoll, Caroline Islands. (**D**) Archeological provenance of analyzed samples in Taumako, Solomon Islands. (**E**) Archeological provenance of analyzed samples in Emae, Vanuatu.

As the last great cycle of human expansion in history, the peopling of Remote Oceania involved the discovery of thousands of uninhabited islands during pulses of rapid expansion and periods of cultural and linguistic differentiation during long pauses of settlement (*1*, *2*). Most western and central Pacific islands were first settled during the Lapita period [3300 to 2900 before the present (B.P.)] by seafarers from Island Southeast Asia and Near Oceania (*3*, *4*). Two thousand years later, thanks to the development of advanced sailing technology and navigational methods such as the large ocean-going double-hulled sailing canoes and the so-called star and wind compasses (*5*, *6*), the Polynesians reached islands further east and quickly settled and thrived on the more distant islands of Hawai’i, Rapa Nui (Easter Island), and Aotearoa (New Zealand), the apexes of the “Polynesian triangle” (Fig. 1A).

Across the western Pacific, where most islands were initially settled from 3000 to 2700 B.P., a set of about 18 societies also stand out for their distinctive Polynesian languages and sociocultural organizations (*7*–*9*). Such communities, collectively known as the Polynesian Outliers, indicate yet another large-scale migratory process and a set of long-distance interarchipelago contacts that remain to be investigated. Language phylogenies, oral traditions, and the analysis of ethnographic, archeological, and archeogenetic data suggest that the Polynesian Outliers are linguistically, culturally, and biologically related to a West Polynesian homeland (*9*–*13*). However, the material evidence of such migrations remains extremely rare, and where evidence is found, it is often unconfirmed. Apart from the few excavations and well-dated contexts (*14*–*17*), a main limitation for detecting Polynesian arrivals in the Outliers is often related to the adoption of material items and technologies by Polynesian-speaking populations from their immediate neighbors. Moreover, overseas voyages and interactions with neighboring southern Oceanic populations are well attested in the oral traditions of Polynesian Outliers (*18*–*21*), which are also revealed by a multitude of shared linguistic and cultural features (*22*–*26*). Although these multiple levels of interactions and migratory events have played an important role in the development and the transformation of Polynesian Outlier societies over time, hard evidence of such external contacts from well-dated archeological contexts remains very scarce (*13*, *27*–*29*).

Provenance studies have revealed the antiquity and the extent of long-distance voyaging among Pacific Islands societies in the past, especially during the Lapita period and following the eastward expansion of the Polynesians (*30*). The transfer of goods between very distant places is assumed to have played a role in maintaining social ties and political alliances between related communities, during phases of expansion and within newly settled regions (*31*–*34*). Here, we present a high-resolution geochemical fingerprinting of artifacts from multiple Polynesian Outliers and unambiguously identify the geological origins of these artifacts. Our study is geographically large scale and required the use of large databases to consider a massive area of our planet, much larger than other similar studies. We provide hard evidence of long-distance voyaging and interisland contacts between the Polynesian Outliers and multiple islands in the Pacific. Exotic materials transported overseas are key to understanding interisland mobility, and we use it here as a proxy to document the origins and timing of westward Polynesian migrations. Our analyses also provide critical information to assess interisland contacts between the newly settled Polynesians and neighboring societies in the western Pacific during the last millennium CE.

## RESULTS

Here, we present new evidence of interisland voyaging to the Polynesian Outliers based on the geochemical sourcing of stone artifacts from three of these islands: Emae in the Shepherd Islands of Vanuatu, Taumako in the Duff group of the Solomon Islands, and Kapingamarangi in the southern Caroline Islands of the Federated States of Micronesia (Fig. 1). We analyzed a total of 14 artifacts, including 8 adzes or adze fragments, 4 flakes, and 2 oven stones (table S1). The artifacts from Emae were collected in the eastern part of the island, where Polynesian-speaking communities historically settled (*36*). While two adzes were found in subsurface contexts, one adze fragment and an obsidian flake were excavated from archeological layers dated to 1565 to 1709 CE and 1565 to 1670 CE (figs. S1 and S2). In addition, we analyzed six geological samples from Emae because no data were available in the literature. From Taumako, we analyzed four complete basalt adzes and one flake associated with the resharpening of a polished adze (fig. S3). Among these, only two adzes were found in stratified deposits associated with the initial settlement of the Kahula site (*17*), which is dated to 1296 to 1472 CE (fig. S4). From the Touhou islet of Kapingamarangi, excavated by Leach and Ward (*37*), we analyzed three flakes of dense volcanic rocks and two cobbles of vesicular basalt identified as oven stones (fig. S5). On the basis of the established chronostratigraphic sequence, two artifacts were in association with deeper cultural deposits dated to 1258 to 1403 CE and 1433 to 1672 CE, respectively, and other artifacts were found in cultural deposits dated to the 17th century or later (text S1 and fig. S6).

Major elements were measured by inductively coupled plasma atomic emission spectrometry (ICP-AES), trace element by inductively coupled plasma mass spectrometry (ICP-MS), and isotopic ratios by multiple-collector ICP-MS (MC-ICP-MS) (text S2 and tables S2 to S4). The geochemical composition of each artifact was compared with data reported for geological samples from Southeast Asia and the Pacific Islands in the GEOROC and the Pofatu databases (texts S3 and S4) (*38*–*41*).

**Fig. 2.**
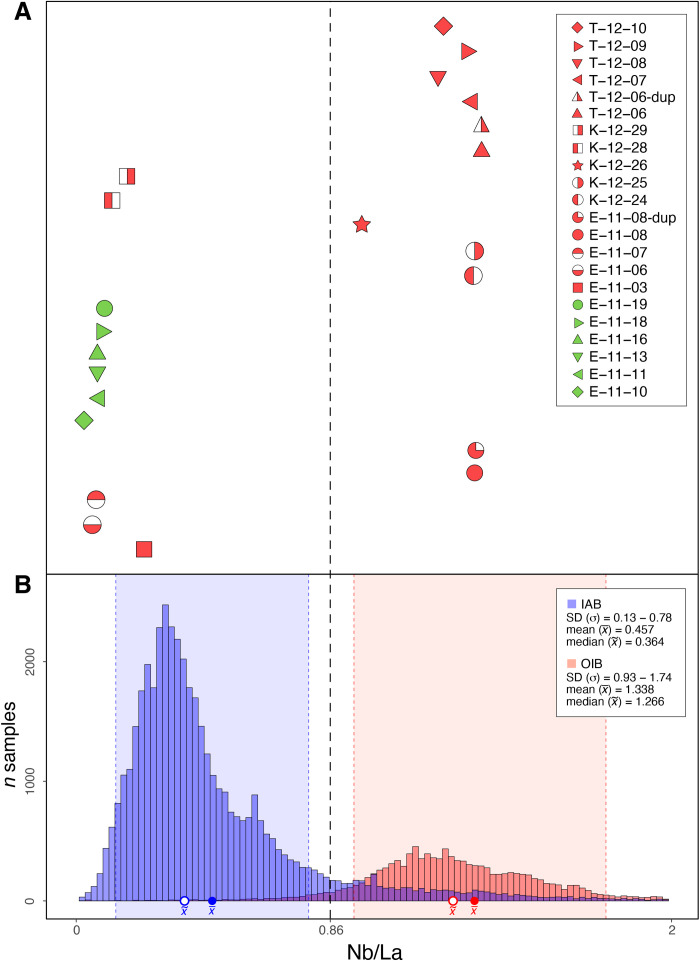
Assessment of geochemical domains based on Nb/La ratio. (**A**) All analyzed samples with the dotted line separating OIB-related samples with higher Nb/La ratios from IAB-related samples with lower Nb/La ratios. (**B**) Values for Nb/La ratios of OIB and IAB are volcanic rocks collected globally from subaerial contexts and compiled in the GEOROC database (*40*, *41*).

Drawing on patterns of chemical variations in geological environments, we demonstrate that artifact compositions are compatible with specific source regions and archipelagos and, when possible, islands and specific quarries. First, we determine which samples are related to island arcs, with possible source islands located west of the andesite line, and which samples are related to intraplate contexts, with possible source islands located east of the andesite line (Fig. 1A). Using a worldwide compilation of data from GEOROC, we show (Fig. 2B) that niobium over lanthanum ratios can be used to separate volcanic rocks from island arcs (IAB) from those present in ocean islands (OIB). Using a threshold of Nb/La < 0.86 for IAB and Nb/La > 0.86 for OIB, we demonstrate that five artifacts in our study were made of island arc materials and nine were made of ocean island materials (Fig. 2A). Primary assessments on the geodynamic origin, rock type, and grouping of analyzed samples are provided in text S3 with a summary in table S6. On the basis of this distinction, we consider 11 possible island arc volcanic chains in the sourcing of our IAB-related samples and 7 possible ocean island chains in the sourcing of our OIB-related samples (Fig. 1A).

Isotopic and elemental compositions of volcanic rocks relate to complex petrogenetic processes and vary in space and time depending on mantle source distributions. Both OIBs and IABs have been documented as chemically and isotopically heterogeneous because of the dynamic evolution of the mantle over time (*42*) and processes such as the recycling of pelagic sediments in the source of subduction-related volcanic rocks (*43*). By mapping the geochemical signatures of available source data from island volcanics in Southeast Asia and the Pacific islands, we can therefore assign a source to each artifact. Thanks to large geochemical datasets documenting a wide range of possible sources from both archeological and geological contexts (*38*, *44*), we are able to ascertain the geological provenance of each artifact, including for islands where the extraction of raw material for artifact manufacture is not yet documented.

### Island arc basalts

**Fig. 3.**
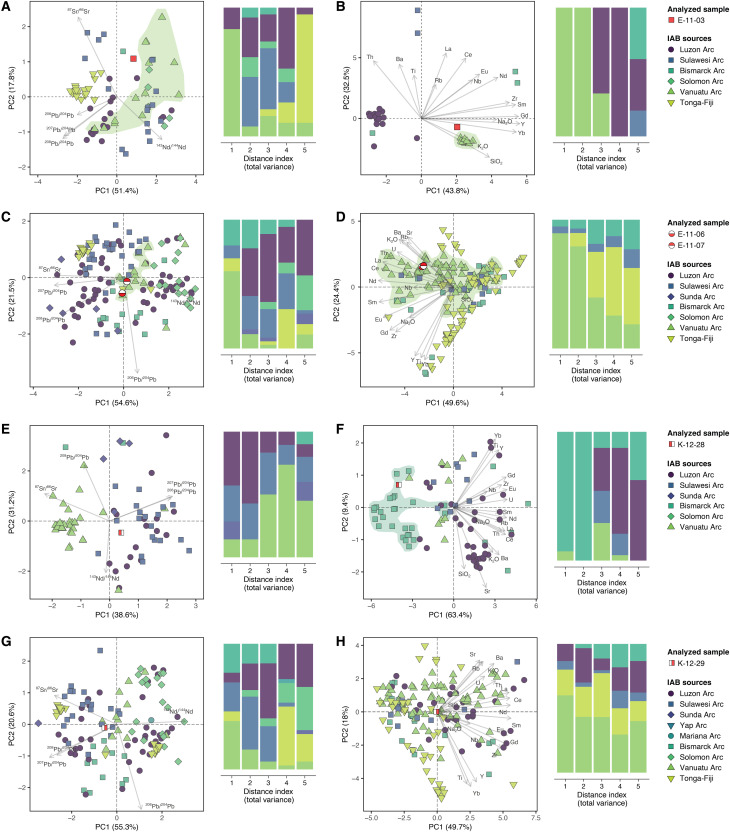
Assignment of IAB-related artifacts to source archipelagos. (**A** to **H**) Principal components analysis (PCA) plots of isotopic compositions (left) and elemental compositions (right). The proportion of explained variance is indicated in parentheses. A measure of distance in all dimensions of the PCA space indicates the degree of compatibility between the artifacts and the reference samples (distance index 1 for the most compatible, and distance index 5 for the least compatible).

Among the five samples with IAB characteristics, three come from Emae Island and two from Kapingamarangi atoll. E-11-03 is a rhyolitic obsidian glass flake from a high-K calc-alkaline series, E-11-06 and E-11-07 are calc-alkaline basalts, and K-12-28 and K-12-29 are a high-K calc-alkaline basalt and a low-K basaltic andesite, respectively (fig. S8A). We find that all three Emae artifacts are made of rocks from the Vanuatu archipelago (Fig. 3, A and C). The classification of E-11-03 as a high-K rhyolitic glass puts severe constraints on the possible source of this sample because this type of volcanic material is quite rare in the region. The only compatible source is an outcrop of obsidian located on the northwest coast of Vanua Lava in the Banks Islands (Fig. 3B and figs. S12 and S13), one of the main sources of obsidian in the prehistory of Vanuatu, although generally associated with the initial settlement period (*45*). The compositions of E-11-06 and E-11-07 are consistent with those of lavas from several islands in Central Vanuatu, including Emae, Efate, Emau, and Tongoa, but the Emae geological samples remain the closest to the two artifacts (Fig. 3D and figs. S12 and S13). The isotopic composition of K-12-28 does not correspond to the signature of any specific arc (Fig. 3E and fig. S11), but its geochemical signature fits with some eastern New Britain volcanics (Fig. 3F), especially the low-K series of the Sulu range and of the Ulawun volcano on the north coast of New Britain (figs. S12 and S13) (*46*, *47*). The isotopic composition of K-12-29 is compatible with several islands in the Bismarck, Luzon, Solomon, Sulawesi, and Vanuatu arcs (Fig. 3G and fig. S11). While several geological samples from the Banks Islands in Vanuatu are among the most similar to K-12-29 (Fig. 3E and fig. S13), basalts of Cebu Island in the Philippines (Luzon Arc) make a better fit with its high-K calc-alkaline series character (fig. S13) (*48*, *49*). Given the untransformed nature of the artifact, we consider it unlikely that the transportation of this stone fragment is anthropogenic. We hypothesize that it was carried in drifted tree trunks, a phenomenon observed by inhabitants of the atoll (*37*). Drifted wood from the Philippines might reach Kapingamarangi when carried by average westerly winds during the northern hemisphere summer and fall, especially under El Niño conditions (*50*).

### Ocean island basalts

**Fig. 4.**
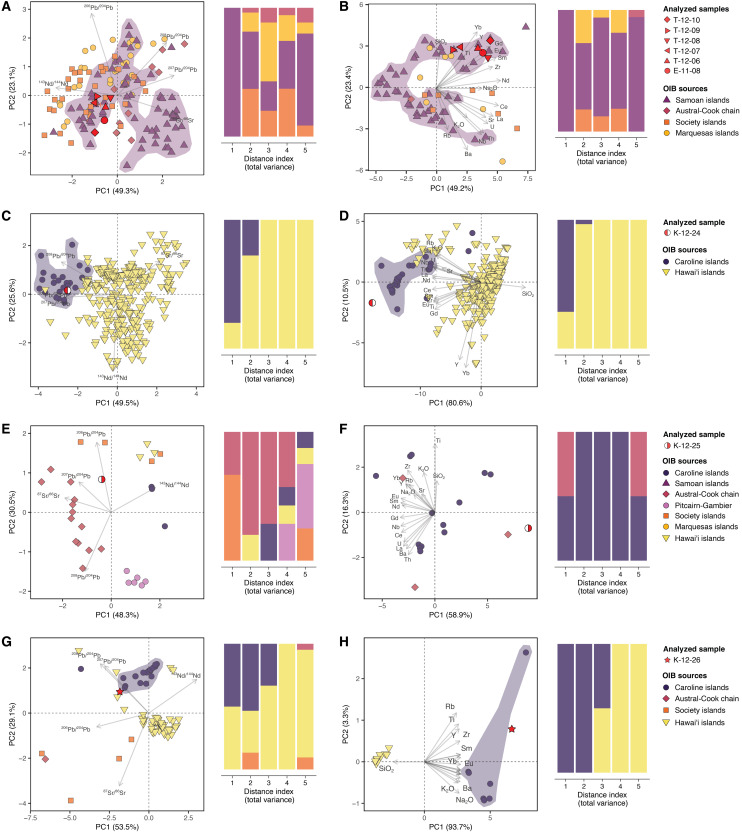
Assignment of OIB-related artifacts to source archipelagos. (**A** to **H**) PCA plots of isotopic compositions (left) and elemental compositions (right). The proportion of explained variance is indicated in parentheses. The measure of distance in all dimensions of the PCA space indicates the degree of compatibility between analyzed artifacts and reference samples (distance index 1 for the most compatible, and distance index 5 for the least compatible).

Archeological samples with OIB characteristics were collected on the three islands (five on Taumako, one on Emae, and three on Kapingamarangi). They all belong to the alkaline series, as shown in the total alkali versus silica diagram (fig. S8B). Most of these are alkali basalts with high Na_2_O contents, but the three Kapingamarangi artifacts were made of a silica-poor basanite (K-12-24), a picrobasalt (K-12-25), and a basanite (K-12-26). E-11-08 and all adze materials from Taumako (T-12-06 to T-12-10) are clearly associated with the Samoan hot spot (Fig. 4A), with their high ^87^Sr/^86^Sr ratios at intermediate ^143^Nd/^144^Nd compared to other OIB sources (fig. S15) (*51*). As a whole, these artifacts have a chemical composition consistent with the Taputapu volcanics on the west side of Tutuila Island, American Sāmoa (*52*), and especially with the 21-ha fortified quarry complex of Tatagamatau (Fig. 4B, figs. S16 to S20), which was first reported by ethnographer P. Buck based on local oral traditions and later surveyed and excavated by archeologists (*28*, *53*–*55*). On the basis of oxide and trace element contents (fig. S18), different subsources can be identified: T-12-07 and T-12-09 have more affinities with the lower part of the main quarry and the adjacent streambed characterized by lower Sr, Rb, and Zr contents and higher Ti/Zr ratios. Other artifacts (E-11-08, T-12-06, T-12-08, and T-12-10) match quarries 1 and 2 in the main fortified Tatagamatau complex, which exhibits higher Sr, Rb, and Nb contents and lower TiO_2_/Fe_2_O_3_ and Ti/Zr ratios. On the basis of oxide contents (fig. S18), the chemical signatures of E-11-08, T-12-06, and T-12-08 match that of samples collected among the workshops located within the Tatagamatau complex, while that of T-12-07, T-12-09, and T-12-10 fit the signature of samples collected among the domestic workshops in the neighboring Malaeloa valley (*56*, *57*).

Artifacts previously sourced to Tatagamatau quarry can also be associated with both subsources (fig. S19). Three artifacts can be sourced to the exact same subsource as samples T-12-07 and T-12-09: sample 6 from the Moturakau rock shelter in Aitutaki (southern Cook Islands) (*58*, *59*), sample COOK-1 from the Anai’o domestic site on Ma’uke (southern Cook Islands) (*28*, *60*), and sample_707 from the Heketa site on Tongatapu in Tonga (*61*). All three artifacts are associated with earlier archeological contexts dated to the late 13th and early 14th century CE. Three other artifacts can be sourced to the exact same subsource as samples E-11-08, T-12-06 and T-12-08, and T-12-10: sample 5 from the Moturakau rock shelter in Aitutaki (southern Cook Islands) (*58*, *59*), sample TOK-A2 from the Atafu atoll in Tokelau (*28*, *62*), and sample 2009-369 from the Tangatatau rock shelter on Mangaia in the southern Cook Islands (*33*, *63*). These artifacts are associated with later archeological contexts dated to the late 14th century up until the 16th century CE.

Turning to the Kapingamarangi artifacts, we find that the isotopic and chemical composition of K-12-24 is highly compatible with that of lavas from the neighboring Caroline Islands (Fig. 4, C and D, and fig. S15). Although Ponape (Pohnpei) geochemically fits (fig. S16), the very low SiO_2_ content of K-12-24 suggests an origin in the low-silicate basalts from Kosrae (fig. S20) (*64*–*66*). The isotopic signature of K-12-25 is not entirely compatible with a particular island chain (Fig. 4E and fig. S15), and its assignment to a specific source is pending on the acquisition of more isotopic data. However, Pb isotopic data exclude the possibility of a source in Hawai’i (fig. S15A), and geochemical data show that K-12-25 is compatible with tholeiitic basalts in Chuuk Island in the Caroline group (Fig. 4F and figs. S16 and S20) (*67*). The isotopic composition of K-12-26 fits with both Hawai’i and the Caroline Islands (Fig. 4G and fig. S15), but an origin in the Caroline Islands seems more consistent with its geochemical features (Fig. 4H). Although K-12-26 is highly depleted in Cs, Rb, and Ba, possibly because of weathering or subsurface alteration processes, its geochemical signature is overall compatible with those of Ponape and Kosrae basanites (figs. S16 and S20) (*65*, *66*).

## DISCUSSION

The settlement history of the Polynesian Outliers has been debated for more than a century. For decades, linguists, ethnographers, and culture historians have questioned whether the Polynesians of the western Pacific could represent relict settlements, marking the trail of early west-to-east migrations toward the central Pacific area (*68*, *69*), or whether they had moved from that region during later east-to-west migrations (*7*, *70*). While historical linguistics and archeological investigations have favored the latter hypothesis with an origin in West Polynesia (*9*, *11*, *14*, *17*), recent work based on limited linguistic and genomic evidence (*71*, *72*) has claimed an origin of eastern Polynesians in northern Outlier islands. Last, computer simulations have revealed that drift voyages of canoes from the central Pacific area could account for the Polynesian settlement of most Outlier islands (*73*).

### Interactions with western Polynesia and the central Pacific area

Our detection of Samoan adzes from the Tatagamatau quarry complex in the archeological record of both Emae and Taumako provides insight into the origins of the Polynesian outlier societies in western Polynesia. The Tatagamatau quarry was first reported in 1930 by ethnographer P. Buck (*53*) based on local oral traditions that mentioned a main procurement site for the production of stone adzes. Later on, the archeological remains associated with the quarry were investigated by archeologists H. Leach and D. Witter, who surveyed and described partially fortified outcrops and stone knapping workshops distributed over a total surface of more than 21 ha (*54*, *55*). The stone knapping workshops in the neighboring valley of Malaeloa were investigated more recently by E. Winterhoff (*56*, *57*), who identified work floors on a total surface of about 14 ha. Previous studies have shown that Tatagamatau adzes were among the most disseminated items in association with the Polynesian expansion across western (*28*, *74*) and eastern Polynesia (*33*, *75*, *58*). Exported artifacts associated with the lower part of the Tatagamatau quarry are found in archeological contexts dated to the late 13th and early 14th century CE in the southern Cook Islands and Tonga, and artifacts associated with the main fortified Tatagamatau complex are associated with later contexts dated to the late 14th century up until the 16th century CE in the southern Cook Islands, Tokelau, and Tonga (*28*, *33*, *58*–*63*). Our assignment of Taumako and Emae adzes to both subsources may suggest that migratory movements toward the Outliers relate to the same bursts of long-distance mobility than those that led to the settlement of East Polynesia. This confirms previous conclusions based on the sourcing of Tatagamatau artifacts (*28*). T-12-06 (artifact 78.76) and E-11-08 (artifact 18-812) are fragments of large blades described as ceremonial adzes, which were typically passed on from generation to generation within chiefly lines in multiple Polynesian societies (*76*, *77*). Despite the fact that both specimens were recovered from surface contexts, we argue that these are likely ancestral tokens transported early on and kept for a considerable amount of time before they were discarded. These can therefore be used as proxies to estimate the origin of Polynesian immigrants within the western Polynesian sphere. A similar argument was made about a group of adzes and a stone image of a fish deity made of fine-grained basalt found in the Polynesian Outlier of Tikopia (Southeast Solomon Islands), which were reported to be petrographically and typologically similar to those of Samoa (*14*, *76*). While accidental landfalls from west Polynesian islands are possible for several Outliers and are documented in several oral traditions and historical accounts (*78*, *79*), the transportation of socially valued items such as adzes made in the Tatagamatau quarry rather suggests carefully planned voyages from West Polynesia to the Outliers.

### The Polynesian outliers in regional exchange networks

The other analyzed artifacts are more likely to represent interactions focused on the distribution of resources locally unavailable, namely, obsidian on Emae and fine-grained basalt and oven stones on Kapingamarangi. The Vanua Lava obsidian flake on Emae (E-11-03) was discarded in a midden mound dated to the 16th century. Obsidians from Vanua Lava have been found as an imported material in the archeological record of several islands within the archipelago (*80*), however not as yet found south of the Banks Islands in post-Lapita contexts. Our results therefore provide evidence of such imports from north to central Vanuatu during the last millennium CE. Obsidian artifacts from the same area were also reported in archeological contexts dated to the last two millennia on Taumako and Tikopia, two Polynesian outliers of the Solomon Islands (*22*, *27*, *29*, *81*). Evidence such as this supports the idea of multiple interactions between these two Outliers, Emae, and the Banks Islands until European contact, as suggested by oral traditions, historical records, and shared material culture items (*17*, *20*, *26*, *82*, *83*).

The source assignment of K-12-24 and K-12-26 will be more convincing once more isotopic data are available for the Caroline Islands. The transportation of stone materials from Kosrae and Ponape Islands to Kapingamarangi supports the existence of a sphere of high mobility between the eastern Caroline Islands and the northern Outliers of Nukuoro and Kapingamarangi. Such interactions have been suggested based on similarities in material culture (shell adzes and fishhooks) and a number of linguistic features and loan words that were shared at a later stage of the cultural sequence (*15*, *37*, *84*).

Last, our tentative assignment of K-12-28 to a source in mainland New Britain illustrates a case of long-distance interaction in the western Pacific that is unseen for the late pre-Contact period. Because sporadic two-way voyages and contacts between the Polynesian atoll of Luangiua (Ontong Java) and Kapingamarangi have been documented for late pre-European times (*20*, *85*, *86*), we hypothesize that transfer between New Britain and Kapingamarangi may have occurred down the line. Cultural contacts between the Caroline Islands and several archipelagos of the southwest Pacific have been suggested to account for the distribution of several technological features, such as Terebridae and Mitridae shell adzes and backstrap loom, which are archeologically and ethnographically attested in the Polynesian Outliers and in several islands of the Caroline, the Admiralties, the Solomon, Santa Cruz, and Vanuatu (*15*, *87*–*89*).

**Fig. 5.**
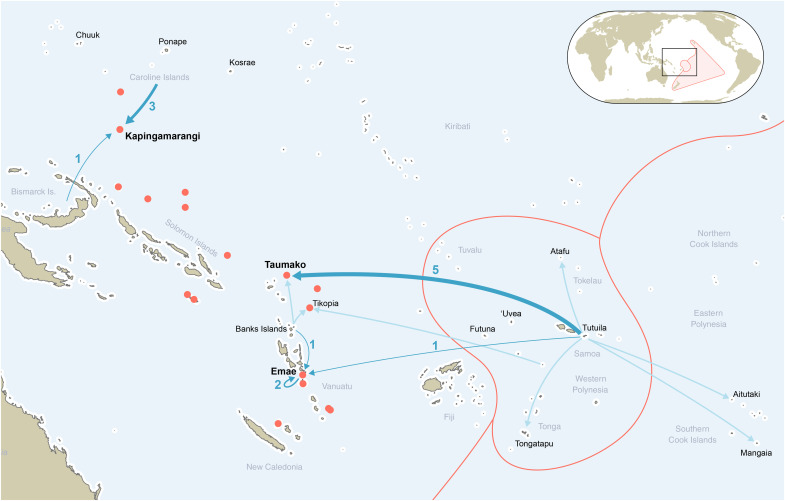
Long-distance interactions involving Polynesian populations in the southwestern Pacific during the last millennium as highlighted by geochemical sourcing studies. Our results are shown in dark blue and previous results (*27*–*29*, *33*, *58*, *61*, *62*) in light blue. The Polynesian Outliers and the limits of western and eastern Polynesia are shown in red.

In summary, our study demonstrates the navigational skills of Pacific Island societies before European contact and emphasizes patterns of mobility in relation to settlement and post-settlement phases in the Polynesian Outliers. The transfer of Samoan adzes produced in the same quarry on the island of Tutuila in two different Outlier islands supports the view that the Outliers were settled from the West Polynesian homeland. We also highlight patterns of interaction between the Polynesian Outliers and neighboring islands that are parallel to many regional pre-Contact exchange networks documented in the Massim, the Vitiaz Strait, the Santa Cruz Islands, and northern Vanuatu (*90*–*93*). These patterns of long-distance interaction were map onto shared material culture items, between the Banks Islands, Emae, Taumako, and Tikopia; between the Caroline Islands, Kapingamarangi, and Nukuoro; and between the Caroline Islands, the Solomon Islands, and Vanuatu (Table 1 and Fig. 5). Although the role played by Polynesian populations in the spread of such cultural features during the last millennium can only be speculated at this point, our geochemical investigation suggests an overall reappraisal of long-distance mobility at a time when Polynesians were sailing across the western Pacific.

**Table 1.** Summary of artifact source assignments.

Sample	Island	Assigned source based on isotopic composition	Assigned source based on elemental composition (island group:island)	Most compatible source samples and bibliographical reference	Comments
**E-11-03**	Emae	Vanuatu or Luzon Arc	Vanuatu:Vanua Lava (Ambek)	ANU9006, ANU9009, ANU9008 (*45*)	No isotopic data available for the obsidian sources of North Vanuatu.
**E-11-06**	Emae	Vanuatu or Bismarck or Fiji-Tonga	Vanuatu:Emae	E-11-11 (this paper)	
**E-11-07**	Emae	Vanuatu or Bismarck or Fiji-Tonga	Vanuatu:Emae	E-11-11 (this paper)	
**E-11-08**	Emae	Samoan Islands	Samoa:Tutuila:Tatagamatau	KC-05-19, KC-05-14 (*32*)	
**T-12-06**	Taumako	Samoan Islands	Samoa:Tutuila:Tatagamatau	KC-05-19, KC-05-14 (*32*)	
**T-12-07**	Taumako	Samoan Islands	Samoa:Tutuila:Tatagamatau	KC-05-19, KC-05-14 (*32*)	
**T-12-08**	Taumako	Samoan Islands	Samoa:Tutuila:Tatagamatau	KC-05-19, KC-05-18 (*32*)	
**T-12-09**	Taumako	Samoan Islands	Samoa:Tutuila:Tatagamatau	KC-05-19, KC-05-14 (*32*)	
**T-12-10**	Taumako	Samoan Islands	Samoa:Tutuila:Tatagamatau	KC-05-19, KC-05-14 (*32*)	
**K-12-24**	Kapingamarangi	Caroline Islands	Caroline:Kosrae or Caroline:Ponape	34227 (*64*); 120903-KOS13-4 (*65*); 1867349,1867355 (*66*)	
**K-12-25**	Kapingamarangi	No clear match	Caroline:Chuuk	143075, 143077 (*67*)	Little isotopic data in support of Caroline Islands but no other trend fits. K-12-25 is compatible with tholeiitic basalts in Chuuk Island in the Caroline group.
**K-12-26**	Kapingamarangi	Caroline Islands	Caroline:Kosrae or Caroline:Ponape	1175178 (*65*); 1867324, 1867344 (*66*)	Little isotopic data in support of Caroline Islands but no other trend fits. K-12-26 is compatible with Ponape and Kosrae basanites.
**K-12-28**	Kapingamarangi	No clear match	Bismarck Arc:New Britain:Sulu Range or Bismarck Arc:New Britain:Ulawun volcano	13436-2649, 13436-2654A, 13423-E5/11 (*46*, *47*)	Little isotopic data available for the Bismarck Arc with the range of ^206^Pb/^204^Pb values fitting K-12-28.
**K-12-29**	Kapingamarangi	No clear match	Philippines:Cebu	144138-KS094 (*48*, *49*)	Isotopic and geochemical signature of K-12-29 fitting Luzon Arc, Sulawesi Arc, Vanuatu Arc, and Tonga-Fiji, but comparable high-K calc-alkaline series only in Luzon Arc (Cebu).

## MATERIALS AND METHODS

### Study design

#### Summary of artifacts provenance

Surveys and excavations on Emae Island were carried by A.H., S.B., E.W., and I.P. during two field seasons in 2018 and 2019. Surveys and excavations on Taumako and Kapingamarangi were carried out by F. Leach and J. Davidson in 1977–1978 (*17*) and by F. Leach and G. Ward in 1979–1980 (*37*). Detailed descriptions of the artifacts, site contexts, and stratigraphy are given in the Supplementary Materials and include the contextual data and available radiocarbon dates (text S1 and figs. S1 to S6).

#### Sampling and sample preparation

We processed and analyzed samples from four artifacts from Emae (Shepherd Islands, Vanuatu), five artifacts from Taumako (Duff Islands, Solomon Islands), and five artifacts from Kapingamarangi (Caroline Islands, Federated States of Micronesia). Sampling of Emae artifacts was done through a research agreement provided by the Cultural Council of the Vanuatu Cultural Centre and with the support of traditional landowners on Emae Island. Sampling of Taumako and Kapingamarangi artifacts was done in agreement with the sampling policy of the Archaeology Laboratory of the Museum of New Zealand Te Papa Tongarewa and with the support of the archeologists who led the excavations in the two islands, J. Davidson and F. Leach. Because no geological data have yet been published for Emae, we also measured the geochemical composition of six samples collected in the vicinity of the archeological sites excavated on the island. The same procedure was followed to extract, process, and analyze all artifacts and geological samples. Core samples were extracted using diamond drill bits of 3- to 5-mm diameter. After selection of small chips presenting no alteration, all core samples were crushed and reduced to a fine powder using a hand agate pestle and mortar.

#### Major element analysis

Major elements and a selected range of trace elements were determined by C.L. at the Pôle Spectrométrie Océan, Institut Universitaire Européen de la Mer (Plouzané, France) following the analytical procedure of Cotten *et al.* (*94*). Results of the ICP-AES analyses are presented in the Supplementary Materials (table S2) and provide compositions of major elements and 17 incompatible trace elements. A description of the analytical procedure is presented in text S2, and details of analytical accuracy are presented in table S3.

#### Trace elements analysis

A broader range of trace elements was measured by P.G. and C.C. using ICP-MS at the Institut de Physique du Globe de Paris, CNRS (Paris, France) following a modified version of the analytical technique presented by Chauvel *et al.* (*95*). Results of the ICP-MS analyses are presented in the Supplementary Materials (table S3) and provide compositions of 37 trace elements. A description of the analytical procedure and details of analytical accuracy are presented in text S2.

#### Pb-Sr-Nd isotope analysis

Isotopic compositions were measured by P.G. and C.C. using MC-ICP-MS at the Institut de Physique du Globe de Paris, CNRS (Paris, France). One hundred milligram of powdered samples was leached in 4 M HCl. The digestion was performed in Savillex beakers in a mixture of 15 M HNO_3−_ 27 M hydrofluoric acid (HF) at 110°C for a minimum of 48 hours until complete dissolution. The chemical isolation of the three elements follows a modified version of the techniques published by Chauvel *et al.* (*95*). All isotopic ratios (^206^Pb/^204^Pb, ^207^Pb/^204^Pb, ^208^Pb/^204^Pb, ^87^Sr/^86^Sr, and ^143^Nd/^144^Nd) were measured on a MC-ICP-MS Neptune Plus. Results of the MC-ICP-MS analyses are given in the Supplementary Materials (table S4), and details on analytical accuracy and fractionation correction are presented in text S2.

### Source assignment

#### Source material

Geochemical sourcing requires reliable and comparable reference data covering all possible source areas. For that purpose, we used data on archeological quarries and on unexploited natural outcrops to consider all potential sources of volcanic rocks in islands in Southeast Asia and the Pacific. Reference material and source data used to assess the provenance of the artifacts are accessed from two online open-access databases: GEOROC (https://georoc.eu/), a global database containing published chemical and isotopic data on geological materials (*44*), and Pofatu (https://pofatu.clld.org/), a database of geochemical compositions focusing on archeological sources and artifacts (*38*). The Pofatu Database is frequently curated and updated, and the data are available as csv-formatted data files and as SQLite data (cf. https://sqlite.org/appfileformat.html; accessed 19 August 2021) in a Zenodo archive (*39*) and in a GitHub repository: https://github.com/pofatu/pofatu-data. The GEOROC data, which are publicly available as csv-formatted files from the GEOROC website (https://georoc.eu/), were also loaded into the SQLite database for scalable and performant analysis, which was made available in the following GitHub repository: https://github.com/pofatu/georoc-data. The Python library and details on the programmatic access to GEOROC data and usage of the SQLite file are documented by R.F. and available online (*96*). Both curated databases are open-access and are fully accessible to reproduce the study.

#### Principal components analyses

Principal components analyses (PCAs) were used to investigate compatibility between artifacts and sources, using the built-in R functions prcomp and predict (*97*). We computed principal components (PCs) from compatible source data, on which artifacts were then projected. Two PCAs were performed for each artifact or group of artifacts with different variables: one using isotopic ratios (^206^Pb/^204^Pb, ^207^Pb/^204^Pb, ^208^Pb/^204^Pb, ^87^Sr/^86^Sr, and ^143^Nd/^144^Nd) and another one using a set of major and trace element contents (SiO_2_, K_2_O, Na_2_O, Rb, Ba, Th, U, Nb, La, Ce, Nd, Sr, Sm, Zr, Ti, Eu, Gd, Y, and Yb). For each PCA, the first two PCs were plotted, along with the distribution of data over the first five PCs (density plots), as well as a distance index representative of the distance from the artifact to each point within the whole PCA space. After calculating distances between artifacts and source sample coordinates in all dimensions of a given PCA space, weighted mean values were obtained using percentages of variance as weights. The range of weighted mean values was divided into intervals and converted into distance indexes from 1 to 5, based on interquartile ranges (1, lower half of the first quartile; 2, upper half of the first quartile; 3, second quartile; 4, third quartile; and 5, fourth quartile). The closest samples to the artifact (distance index 1) are considered the most compatible and most likely the geological source. Two PCAs were performed for each sample or set of geochemically similar samples: the first one based on isotopic data and the second one based on major and trace element data.

#### Exploration of isotopic and geochemical data

In parallel to PCAs, a general procedure has been followed to investigate the source of each artifact, using biplots of isotopic ratios to visualize Pacific-wide trends (figs. S11 and S15), biplots of oxides, trace elements and trace element ratios to confirm compatibility with islands or groups of islands (figs. S12 and S16 to S19), multielement plots with values normalized to the primitive mantle (*98*), and K_2_O versus SiO_2_ (*99*) and total alkali silica diagrams (*100*) to detect possible sources and narrow down the analysis (figs. S13 and S20).

### Code availability

The computer code used to generate the Bayesian age models are provided in full in the Supplementary Materials, together with information about the program and version used. To ensure full reproducibility, source data and R scripts are available in a GitHub repository (https://github.com/tupuni/polynesianoutliersvoyaging/releases/tag/v1.0) and in the following archive: http://doi.org/10.5281/zenodo.7388196.
