# Supplementary material for: Artifact geochemistry demonstrates long-distance voyaging in the Polynesian Outliers
Source: Sci Adv. 2023 Apr 21;9(16):eadf4487. doi: 10.1126/sciadv.adf4487 (PMC10121159; doi:10.1126/sciadv.adf4487)
Supplement: Supplementary file 1 — Figs. S1 to S20 Tables S6 and S7 Legends for tables S1 to S8 Supplementary Text S1 to S5 [file sciadv.adf4487_sm.pdf]

Science Advances
AAAS

Supplementary Materials for

Artifact geochemistry demonstrates long-distance voyaging in the Polynesian Outliers

Aymeric Hermann *et al.*

Corresponding author: Aymeric Hermann, aymeric.hermann@cnrs.fr

Sci. Adv. **9**, eadf4487 (2023)
DOI: 10.1126/sciadv.adf4487

The PDF file includes:

Figs. S1 to S20
Tables S6 and S7
Legends for tables S1 to S8
Supplementary Text S1 to S5

Other Supplementary Material for this manuscript includes the following:

Tables S1 to S5 and S8

Supplementary Tables

Tables S1-S4. Provenance of samples and descriptive results.
Combined as tabs in Microsoft Excel spreadsheet:

Table S1. "Inventory"
Inventory and provenance of analyzed samples.

Table S2. "ICP-AES data"
Major element compositions (in weight %) measured by ICP-AES at PSO/IUEM.

Table S3. "ICP-MS data"
Trace element compositions (in ppm) measured by ICP-MS at IPGP.

Table S4. "MC-ICP-MS data"
Nd, Sr and Pb isotopic ratios measured by MC-ICP-MS at IPGP.

Table S5. CQL code for the Bayesian model of Kapingamarangi sequence.

Table S6. Sample assignment to geochemical domains.
Embedded in SI 3. General assessment of geochemical domains.

Table S7. Range values for SQL queries.
Embedded in SI 4. Methodology of source assignments.

Table S8. List of SQL queries used to retrieve matching source data.

SI 1. Archaeological contexts

The 14 artefacts analyzed in this study (Table S1) were collected from three islands known as Polynesian outliers. The eastern part of Emae Island (Shepherd Islands, Central Vanuatu) was investigated by A. Hermann, S. Bedford, E. Willie and I. Phillip during two field seasons in 2018 and 2019. Taumako (Duff group, Southeast Solomon Islands) and Kapingamarangi (Eastern Caroline Islands) were surveyed and excavated respectively by Foss Leach and Janet Davidson in 1977-1978 (*17*), and by Foss Leach and Graeme Ward in 1979-1980 (*37*).

Emae (Shepherd group, Vanuatu)

The artefacts from Emae (17°4′00″ S, 168°22′30″ E) include one complete adze, two adze fragments (distal edge), and one complete flake volcanic glass (Figure S1). In terms of petrography this assemblage is rather diverse with different kinds of igneous rocks represented. All artefacts were found in the eastern part of the island, where Polynesian-speaking communities are settled (*36*). Other Polynesian-speaking communities are present in the southern part of Efate, on Fila/Ifira island and Mele/Mwele islet and attest to the settlement of populations from West Polynesia who successfully integrated the cultural hub of central Vanuatu (*26*). Although the exact date of these migrations remains unclear, the continuity in the cultural sequence and the occurrence of pottery in all cultural layers up until the ash deposits related to the nearby Kuwae eruption (*87*, *101*) would indicate that these communities might not have settled the island before the 15th century AD. Two adzes were found in subsurface contexts and were kindly provided for analysis by Nicholas Jacob (artefact 18-811, sample E-11-07) and by Melten Kalo (artefact 18-812, sample E-11-08). The obsidian flake (18-582, sample E-11-03) and the adze fragment (18-164, sample E-11-06) were found in post-Kuwae layers respectively dated to 1565-1709 and 1565-1670 cal AD at 2σ (Figure S2).

A set of six geological samples (E-11-10 to E-11-19) were also collected from boulders in scree areas around Maunga Lasi (eastern volcano of Emae) in order to provide reference material to geochemically characterize the island.

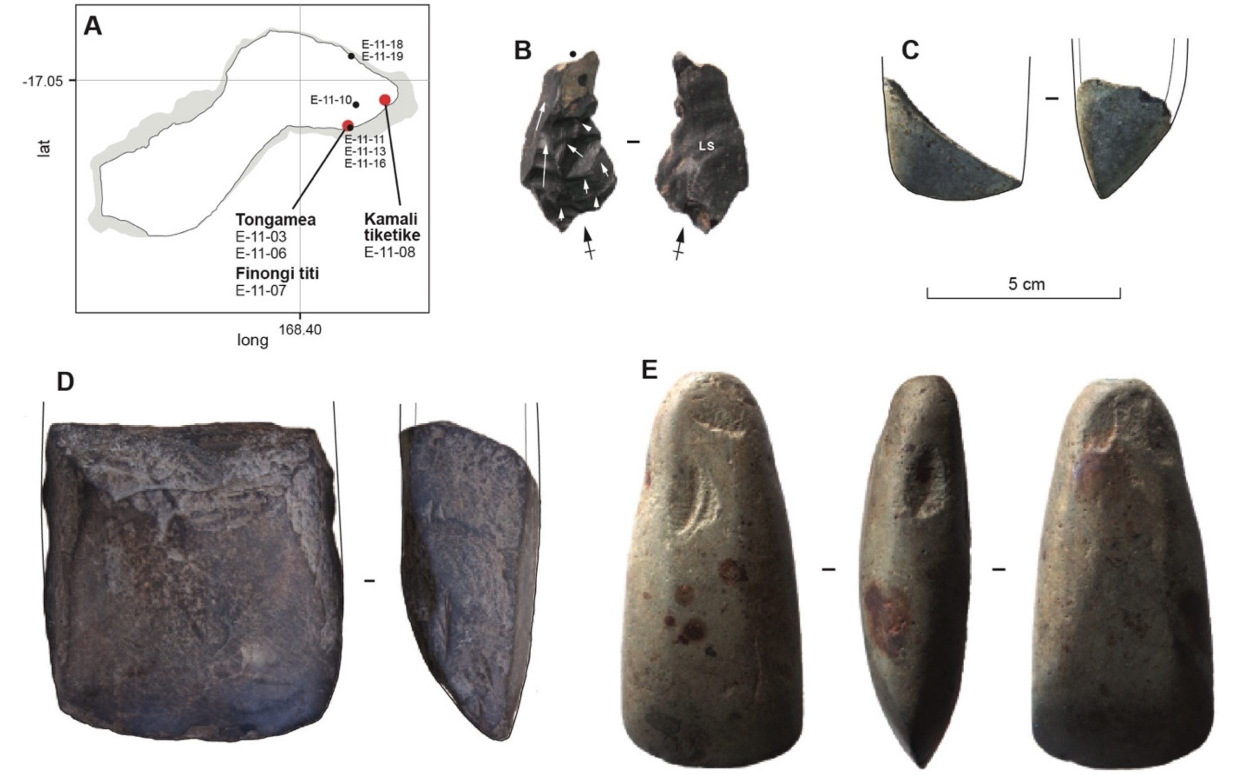

Figure S1. Sampled artefacts from Emae. (**A**) Provenance of sampled artefacts (red dots) and geological samples (black dots on the map) on Emae Island. (**B**) Obsidian flake 18-582 (s E-11-03). (**C**) Distal fragment of polished adze 18-164 (s E-11-06). (**D**) Distal fragment of polished adze 18-812 (s E-11-08). (**E**) Polished adze 18-811 (s E-11-07). Photo and Figure credit: Aymeric Hermann.

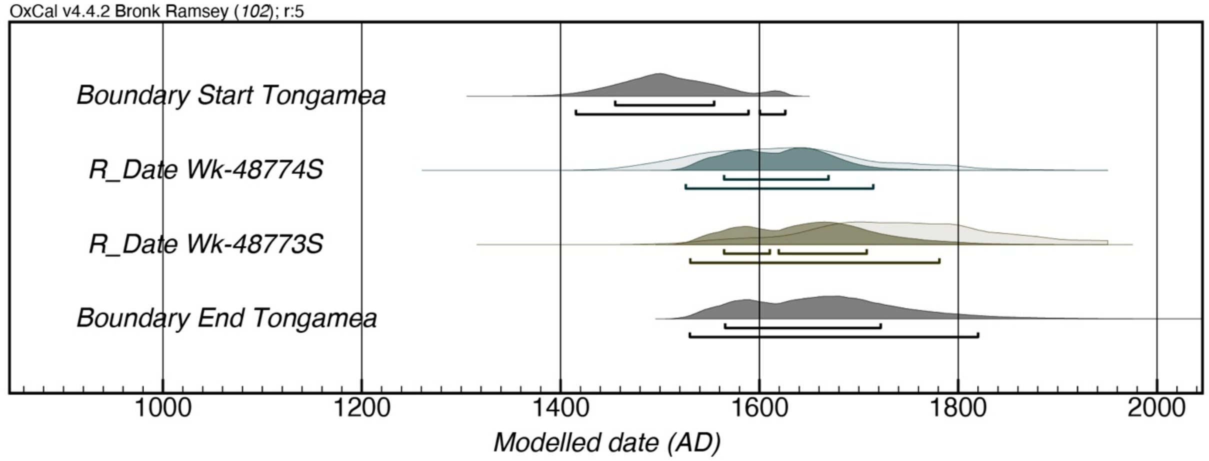

Figure S2. Modelled dates for the upper archaeological layers of Tongamea, Emae. Figure credit: Aymeric Hermann.

Taumako (Duff group, Solomon Islands)

All Taumako (9°52′30.0″ S, 167°10′44.0″ E) artefacts were studied and sampled at the Archaeology Laboratory of the Museum of New Zealand Te Papa Tongarewa (Wellington). Three of them were collected by R. Green in 1972: a flake associated with the maintenance of a polished adze (A57), which was found on the surface of the Kahula site, and two complete adzes discarded after a failed maintenance procedure (A138 and A172), which were found in the archaeological layers of the same site. Two adzes found in surface contexts by local informants were given to F. Leach and J. Davidson during their field season on the island in 1978: one was found in the Kongo area (78.76) and the other in the Kahula area (78.77) (*17*). All adzes were previously sampled (78.76 as sample TAU54, 78.77 as sample TAU48, A138 as sample TAU49, and A172 as sample TAU52) and submitted to both petrographic and geochemical analyses as part of a pioneering study published by Best and colleagues (*28*). Although comprehensive at the time, WD-XRF analyses only allowed to measure oxides and a limited range of trace elements, at the cost of invasive sampling procedures that are visible on the artefacts (Figure S3). Nearly 30 years later, our analyses provide a better coverage of the geochemical composition of these artefacts, especially regarding the isotopic signatures which were not previously documented.

Adzes A172 and A138 (samples T-12-07 and T-12-08) were both associated with the lower occupation layer of the Kahula site. A single unidentified charcoal from this initial occupation was dated by the Australian National University Radiocarbon Laboratory (SUA-116), which provided a Conventional Radiocarbon Age of 530 ± 65 BP (*17*). We calibrated it using the online software OxCal 4.4.2 (*102*) and both IntCal20 Northern hemisphere (*103*) and SHCal20 Southern hemisphere (*104*). Calibrated dates using IntCal20 are slightly older (1296-1472 AD at 2σ) than the ones obtained when using SHCal20, which has a higher probability (79.5%) for 1381-1509 AD at 2σ and a higher probability (66.9%) for 1393-1461 AD at 1σ (Figure S4). Both calibrations suggest an initial occupation of the site before the beginning of the 15th century.

The petrographic observations of H.J. Campbell (GNS Science) indicate that all adzes were made of a similar fine-grained aphyric basalt bearing the same silicate minerals, including olivine and biotite (*17*). Moreover, these artefacts exhibit common typological characteristics which fit with the Samoan type 1 and 2 as defined by R. Green and J. Davidson (*105*).

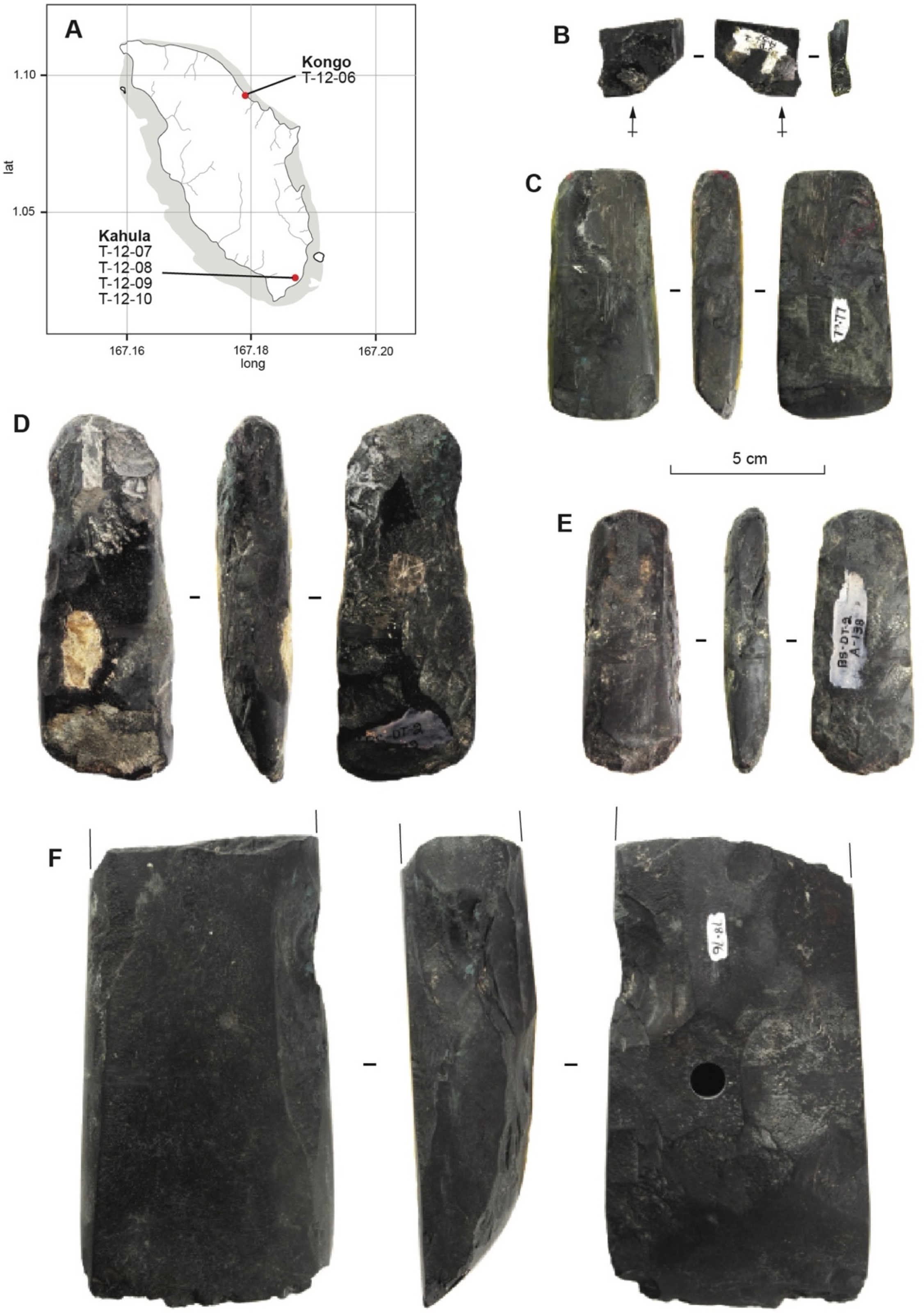

Figure S3. Sampled artefacts from Taumako. (**A**) Provenance of sampled artefacts on Taumako Island. (**B**) Polished adze flake A57 (s T-12-10). (**C**) Polished adze 78.77 (s T-12-09). (**D**) Retouched adze A172 (s T-12-07). (**E**) Modified adze A138 (s T-12-08). (**F**) Distal fragment of polished adze 78.76 (s T-12-06). Photo and Figure credit: Aymeric Hermann.

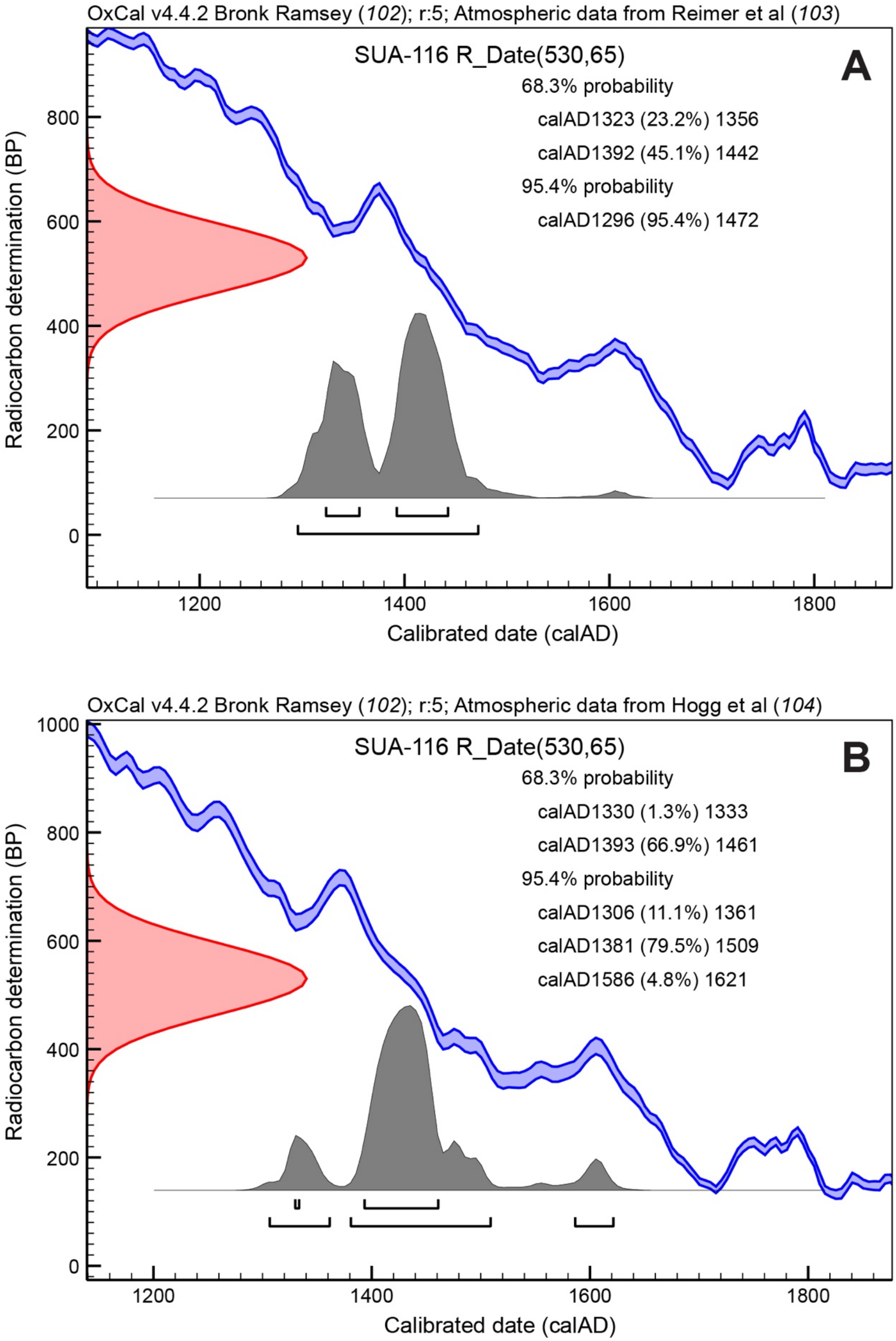

Figure S4. Calibrated age of SUA-116 from the lower level of the Kahula site. (**A**) Calibration using IntCal20 Northern hemisphere (*103*). (**B**) Calibration using SHCal20 Southern hemisphere (*104*). Figure credit: Aymeric Hermann.

Kapingamarangi (Caroline Islands, Federated States of Micronesia)

The Kapingamarangi (1°04′42.0″ N, 154°48′28.0″ E) artefacts were collected in test pits located on the *motu* Touhou, near the lagoon shore at Muri-Harau (KA-2), and near the ocean side at Putau (KA-3). These were studied and sampled at the Archaeology Laboratory of the Museum of New Zealand Te Papa Tongarewa (Wellington). Two are cobbles of vesicular basalt (79.44 and 79.555) identified as oven stones, and the remaining three are flakes or undetermined fragments (79.549, 79.43b, 79.551) of dense volcanic rocks (Figure S5). Petrographic descriptions by G. Mason previously confirmed that all artefacts selected for analysis are volcanic rocks and their archaeological provenance is based on the stratigraphies reported by Leach and Ward (*37*).

All artefacts were found in association with a cultural context, except 79.555, which was recorded in the underlying coral substrate of KA-3, but it is possible that it has been redeposited due to the fluctuating seawater level and could actually be related to the above cultural layer 10. In the interpretation of Leach and Ward, the chrono-stratigraphic sequence of *motu* Touhou is divided in 4 phases, which were established based on a set of 12 radiocarbon dates and three modern coins found at KA-2. A new estimate of the cultural sequence was ran in a Bayesian framework using OxCal 4.4.2 (*102*) and IntCal20 Northern hemisphere (*103*). Based on this, it is possible to estimate the age of the cultural contexts associated with the sampled artefacts (Figure S6). 79.549 (sample K-12-26) is associated in phase 4 with Japanese 10 sen and American 5 cents coins respectively dated to 1942 and 1964. Artefacts 79.44 (K-12-25) and 79.43b (K-12-28) are stratigraphically located between phase 3 and 4 and can therefore be associated to the modelled boundary range of 1641-1945 AD. Artefact 79.551 (K-12-24) is associated with phase 3 which is modelled to 1433-1672 AD. Artefact 79.555 (K-12-29) is associated with phase 1, which is modelled to 1258-1403 AD.

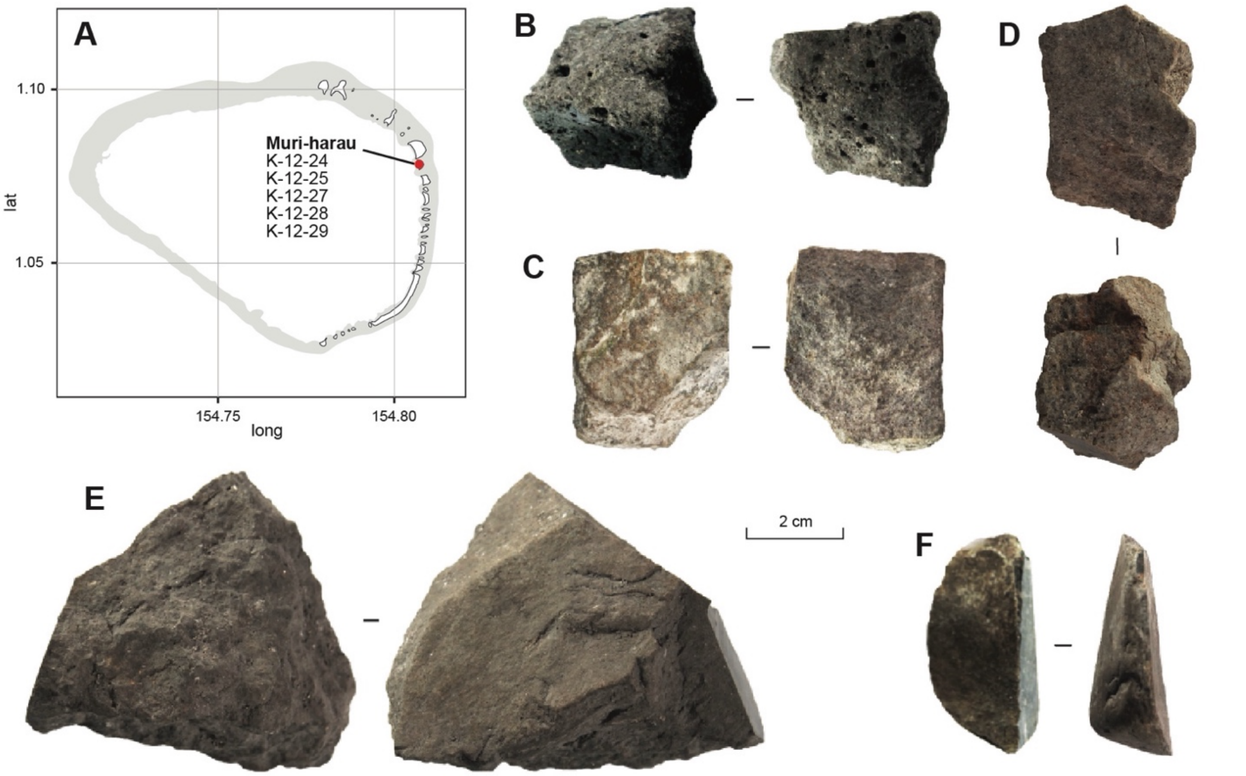

Figure S5. Sampled artefacts from Kapingamarangi. (**A**) Provenance of sampled artefacts on *motu* Tohou. (**B**) Oven stone 79.44 (s K-12-25). (**C**) Flake 79.549 (s K-12-26). (**D**) Undetermined fragment 79.555 (s K-12-29). (**E**) Undetermined fragment 79.551 (s K-12-24). (**F**) Flake 79.43b (s K-12-28). Photo and Figure credit: Aymeric Hermann.

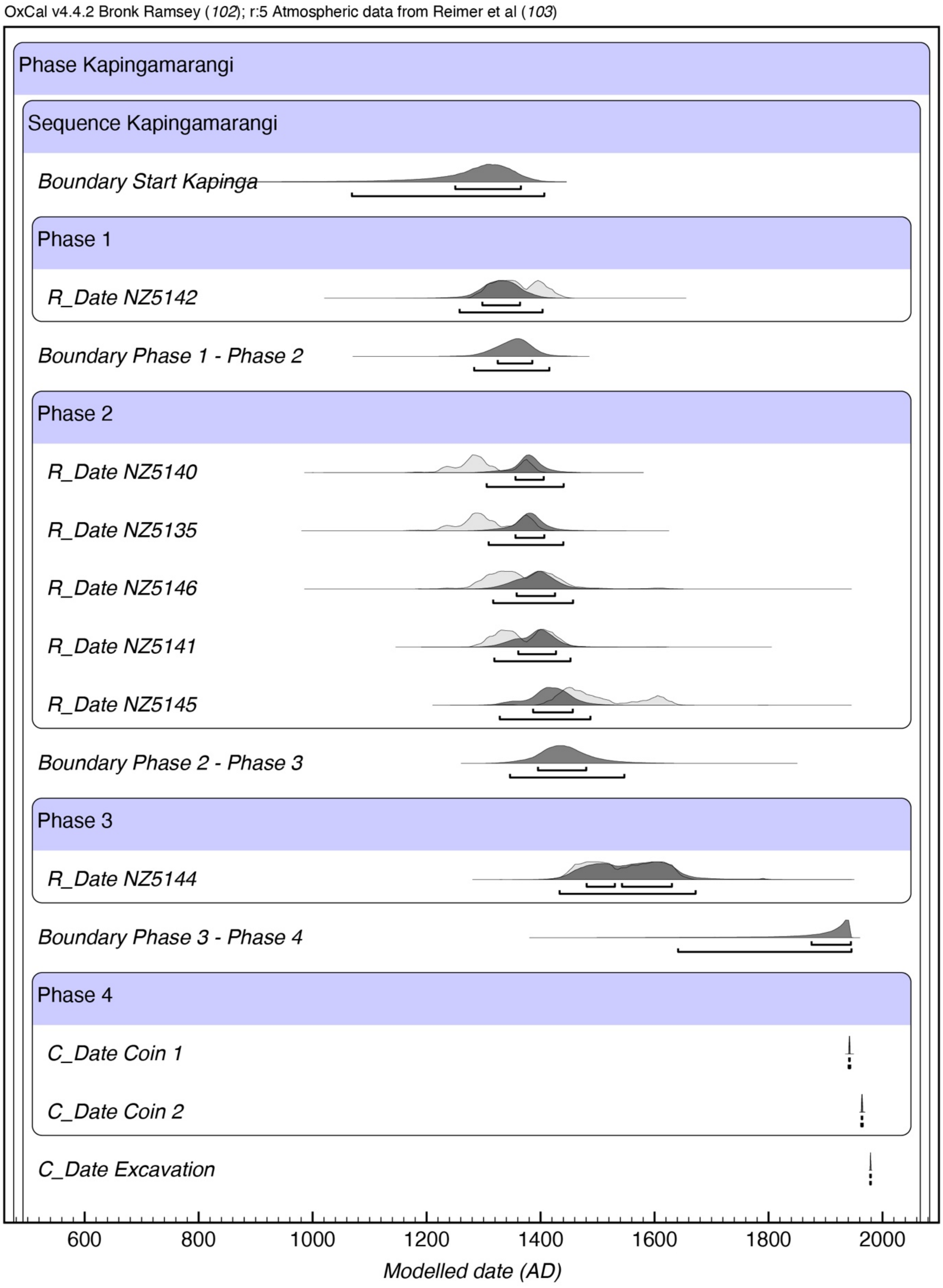

Figure S6. Kapingamarangi cultural sequence based on Leach and Ward (*37*)**.** Figure credit: Aymeric Hermann.

SI 2. Analytical procedures

Major element analysis

Major elements and a limited range of trace elements were determined by C.L. at the Pôle Spectrométrie Océan, Institut Universitaire Européen de la Mer (Brest, France) following the analytical procedure of Cotton and colleagues (*94*). Typically, 250 mg of rock power were dissolved in closed screw-top teflon vessels (Savillex®) at about 90°C for one day using 3 ml of concentrated HF, and 1 ml of concentrated HNO_3. Next, 96 ml of H_3BO_3 aqueous solution (20 g/L H_3BO_3) were added to neutralize the excess HF. All reagents used are analytical grade. Elements were measured by inductively coupled plasma-atomic emission spectrometry (ICP-AES) using a Horiba Jobin Yvon® Ultima 2 spectrometer. The boron included in the solution was used as an internal standard. Calibrations were made using international standards ACE, JB-2, CB2, BELC, CB15 and CB18 and the accuracy and precision of major element data was assessed from the measured concentrations for standards ME and WS-E. Relative standard deviation is ≤ 1% for SiO_2, ≤ 2% for all other major elements, and ≤ 5% for all measured trace elements. Measured values for all samples are presented with international standards, along with details of analytical accuracy (Table S2).

Trace elements analysis

A broader range of trace elements was measured by P.G. and C.C. using Inductively Coupled Plasma Mass Spectrometry (ICP-MS) at the Institut de Physique du Globe de Paris, CNRS (Paris, France) following a modified version of the analytical techniques presented by Chauvel and colleagues (*95*). About 50 mg of powder were precisely weighted and digested in Savillex® vials at 110ºC using first 15M ultrapure HNO_3 for 24h followed by a mixture of 15M HNO_3 27 M HF for a minimum of 5 days. After dissolution, they were diluted in 2% v/v HNO_3 to obtain a dilution factor of 10000 and minimize matrix effects during measurements on the ICP-MS. Measurements were performed using a Quadrupole ICP-MS Agilent 7900, the international reference material BCR-2 as calibration standard and correcting the instrumental drift by standard bracketing. The accuracy and precision of the analytical procedures were assessed by the analysis of 2 total procedure duplicate samples, 2 procedural blanks, and the rock standard BR-24 run as unknown. The relative bias between the measured values and those published by Chauvel and colleagues (*95*)

is generally less than 3%; the relative bias between duplicates is also low (< 3 %), and the total procedural blanks are negligible at less than 0.08 % of the amounts present in the samples. Measured values for all samples and BR-24 are presented in Table S3.

Pb-Sr-Nd isotope analysis

Isotopic compositions were measured by P.G. and C.C. using Multiple Collector Inductively Coupled Plasma Mass Spectrometry (MC-ICP-MS) at the Institut de Physique du Globe de Paris, CNRS (Paris, France). One hundred mg of powdered samples were leached in 4M HCl for one hour, rinsed with MilliQ 18.2 Ω water several times and dried down. The digestion was performed in Savillex® beakers in a mixture of 15M HNO_3-27M HF at 110ºC for a minimum of 48h until complete dissolution. The chemical separation of the three elements follows a modified version of the techniques published by Chauvel and colleagues (*95*). The dried down samples were dissolved in 0.7M HBr and centrifuged. Samples were loaded on columns made of retractable Teflon and filled with anion-exchange AG1-X8 resin; the bulk matrix containing the Sr and the Nd was collected with 0.7M HBr + 2M HCl while the Pb still retained on the resin was collected using 6M HCl. The Pb fraction was passed on the columns again in order to obtain a cleaner Pb fraction. The bulk matrix containing Sr and Nd was loaded on a column filled with cationic AG50W-X8 resin; the Sr fraction was collected with 2M HCl and 3.5M HCl and the REE fraction containing the Nd was collected using 6M HCl. The Sr fraction was further purified using a small column filled with Sr-spec resin to ensure the absence of Rb which can interfere with the measurement of the isotopic ratios $^{87}Sr/^{86}Sr$. The matrix and the Rb were removed with 3M HNO_3, and the Sr was collected using milliQ water. Finally, Nd was isolated from other REE through a chromatography on Ln-Spec resin. The collected Nd fraction eluted with 0.25M HCl did not contain any Sm, element that interferes when measuring the isotopic ratio $^{143}Nd/^{144}Nd$. All isotopic measurements were performed using a MC-ICP-MS Neptune Plus in static mode. Lead isotopic measurements were corrected for mass fractionation using the thallium addition technique (*106*). Strontium isotopic compositions were normalized internally to $^{86}Sr/^{88}Sr = 0.1194$ and neodymium to $^{146}Nd/^{144}Nd = 0.7219$. In addition, the isotopic ratios were corrected for instrumental bias using the standard-sample bracketing technique and running a reference standard every three samples. Although the values of the measured standards (NBS-981 for Pb, NBS-987 for Sr and AMES-Rennes and JNdi for Nd) were slightly different from the recommended values for Pb and Sr, they remained stable throughout the sequence and a correction could be applied using the recommended

values by Jochum and colleagues (*107*). The quality of the data was assessed through total procedural chemistry blanks, duplicates sample analyses and one external rock standard which was subjected to the same protocols as the samples. The blank values correspond to less than 0.05 % of the amounts present in the samples (Pb < 35 pg, Sr < 192 pg, Nd < 50 pg) and these quantities can be considered as negligible. The difference between duplicate samples is less than 0.06 % and the measured isotope ratios of the rock standard BR-24 are consistent with the values recommended by Chauvel and colleagues (*95*). Measured values for all samples and international standards are presented in Table S4.

SI 3. General assessment of geochemical domains

Based on values collected globally, we used niobium versus lanthanum ratios to differentiate between sources in island arcs and in intraplate oceanic islands. We used the GEOROC SQLite database to investigate such global chemical patterns while considering a dataset of Nb/La values under 3 for volcanic rocks collected in subaerial contexts – a total of 44109 samples for Convergent Margins (*41*) and 12387 samples for Ocean Islands (*40*). We found that an intermediary value of 0.86 – between one standard deviation above the mean of island arc materials and one standard deviation under the mean of ocean island materials – can be used to assign a sample to either island arcs (IAB) or ocean islands (OIB). Based on that estimate, 5 artefacts in our study are related to IAB and 9 to OIB (Figure S7).

According to the K_2O versus SiO_2 classification of Peccerillo and Taylor (*99*), all geological samples from Emae are calc-alkaline basalts, as well as the archaeological samples E-11-06 and E-11-07 collected on the same island, while E-11-03 is a rhyolitic obsidian glass flake from a high-K calc-alkaline series. K-12-28 and K-12-29 are respectively a low-K basaltic andesite and a high-K calc-alkaline basalt (Figure S8-A). All archaeological samples related to OIB sources can be classified in the alkaline series. The Taumako adzes and E-11-08 form one group of alkali basalts with high Na_2O content (> 3%), while K-12-24 is a silica-poor basanite, K-12-25 a picrobasalt, and K-12-26 a regular basanite (Figure S8-B). Multi-element patterns also provide useful information to differentiate between geological sources from island arc and from oceanic islands (Figure S9). Enriched OIB have high concentrations of incompatible trace elements and in particular Nb, Ta, La, Ce because they relate to deep mantle melting processes. In contrast, the mafic arc volcanics are depleted in High Field Strength Elements (HFSE), which results in negative Nb-Ta anomalies; they also have positive Pb anomalies.

For analytical purposes, we grouped together the samples that were chemically most similar, based on their geodynamic origin and classification. A summary of primary assessments on the geodynamic origin, rock type, and clustering of each analyzed sample is provided in Table S6.

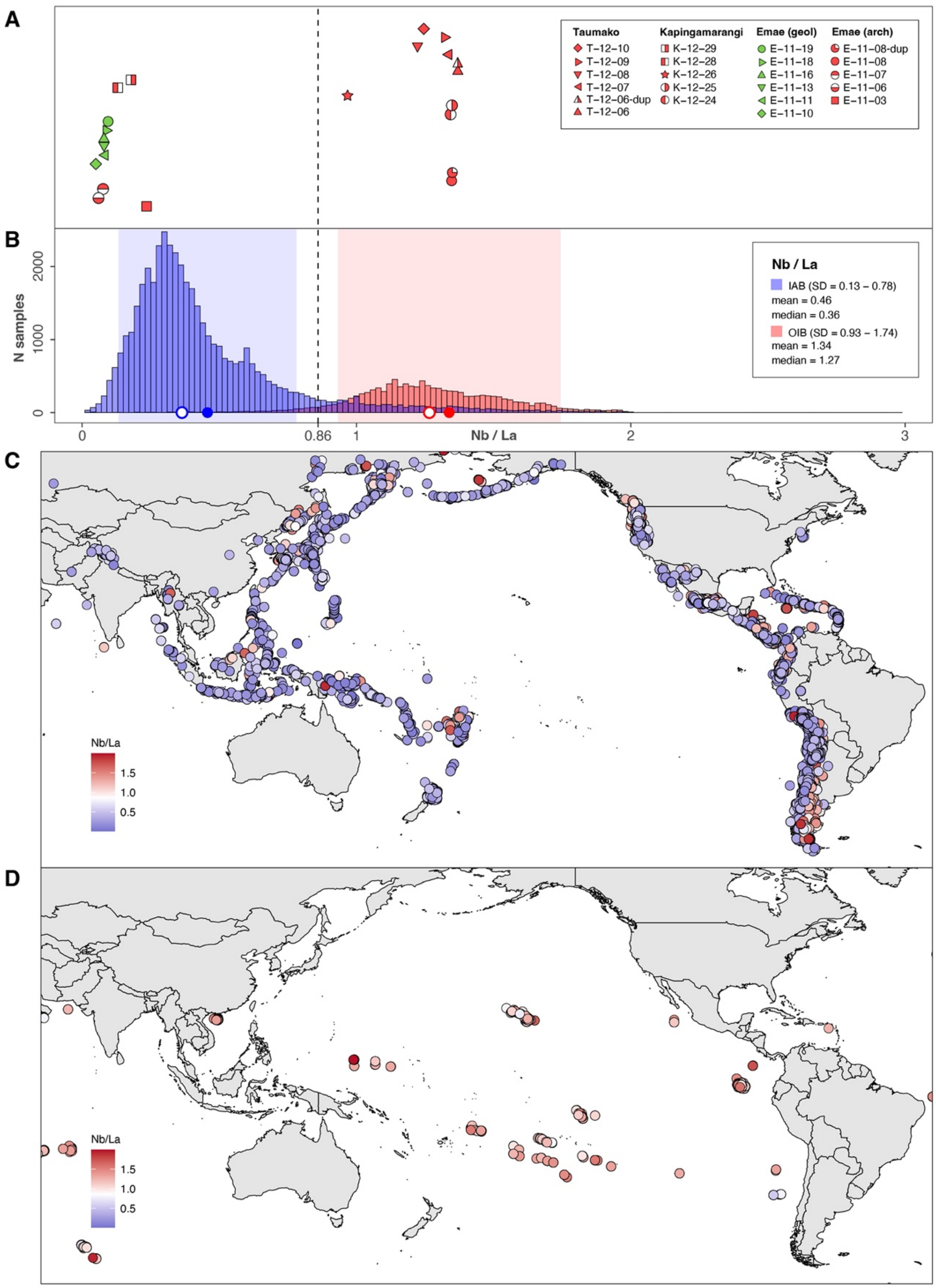

Figure S7. Geochemical domains and sample assignment to Island Arc volcanics et Ocean Island volcanics. (**A** and **B**) Nb/La ratios for all samples in this study, versus Convergent margins and Ocean islands in the GEOROC database. The dotted line separates the two fields at a midpoint between the maximum standard deviation of IAB values and the minimum standard deviation of OIB values. (**C** and **D**) Spatial distribution of reference samples from Convergent margins and Ocean islands in the GEOROC database (*40*, *41*). Figure credit: Aymeric Hermann.

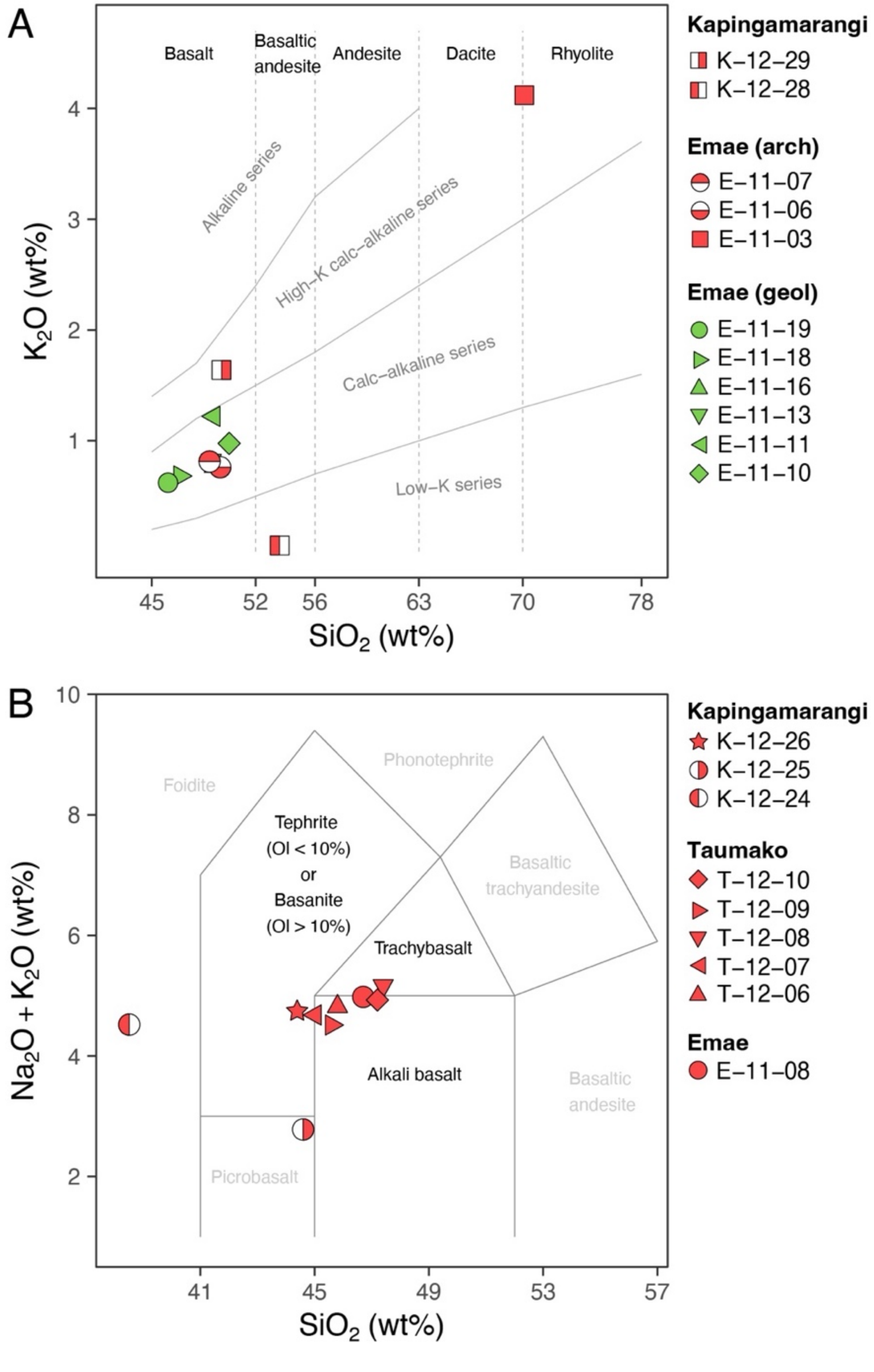

Figure S8. Classification of analyzed samples. (**A**) K_2O versus SiO_2 plot of IAB-related samples, adapted from Peccerillo and Taylor (*99*). (**B**) Total Alkali Silica plot of OIB-related samples, adapted from Le Bas and colleagues (*100*). Figure credit: Aymeric Hermann.

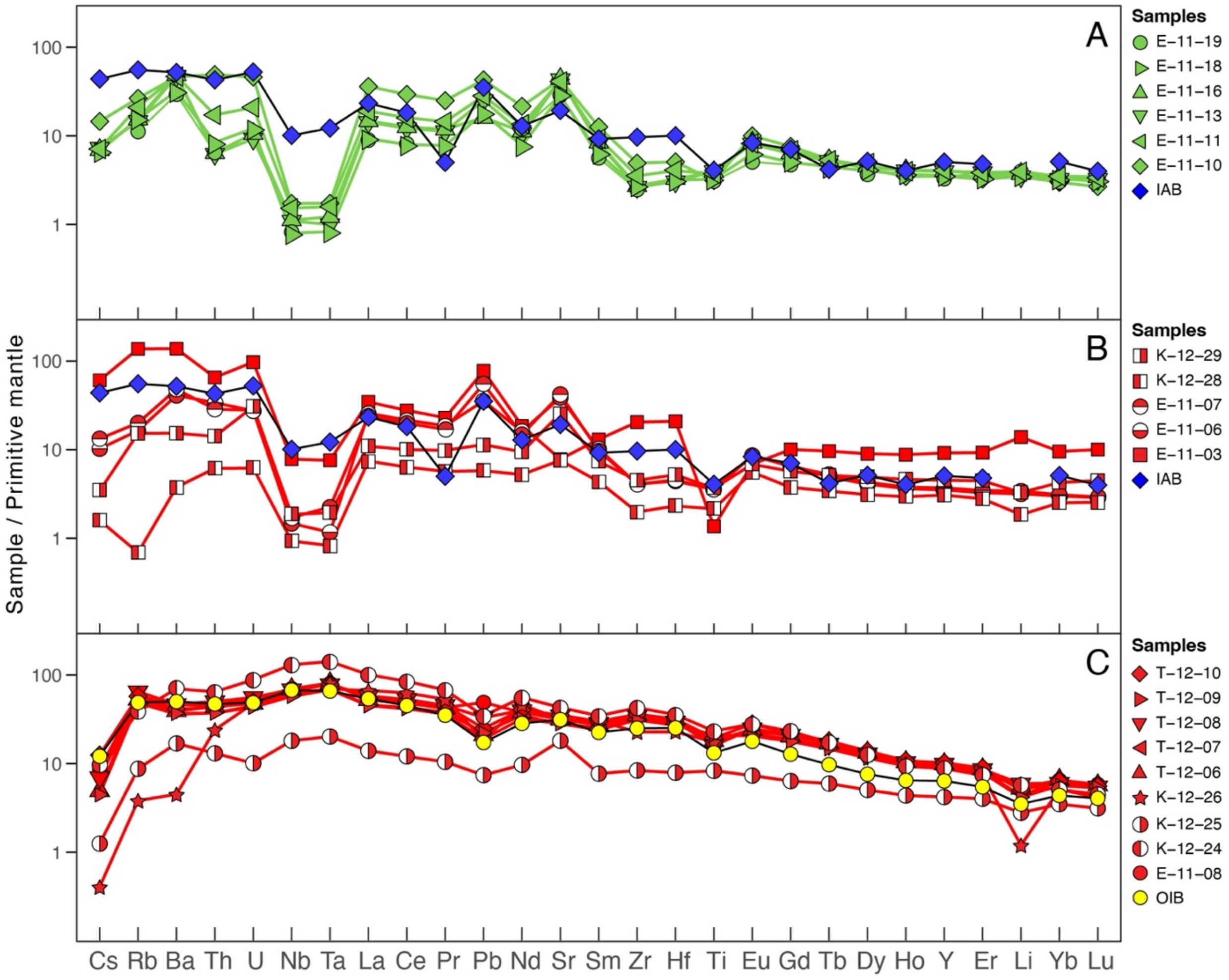

Figure S9. Multielement plots for all samples in this study. (**A**) Emae geological samples. (**B**) Archaeological samples with IAB characteristics. (**C**) Archaeological samples with OIB characteristics. All values are normalized to the Primitive mantle (*98*). Figure credit: Aymeric Hermann.

Table S6. Sample assignment to geochemical domains. Table credit: Aymeric Hermann.

Sample	Island	Geochemical domain	Material	Group
E-11-03	Emae	Island Arc	Rhyolite	1
E-11-06	Emae	Island Arc	Calc-alkaline basalt	2
E-11-07	Emae	Island Arc	Calc-alkaline basalt	2
E-11-08	Emae	Oceanic Island	Alkali basalt	3
E-11-10	Emae	Island Arc	Calc-alkaline basalt	2
E-11-11	Emae	Island Arc	Calc-alkaline basalt	2
E-11-13	Emae	Island Arc	Calc-alkaline basalt	2
E-11-16	Emae	Island Arc	Calc-alkaline basalt	2
E-11-18	Emae	Island Arc	Calc-alkaline basalt	2
E-11-19	Emae	Island Arc	Calc-alkaline basalt	2
T-12-06	Taumako	Oceanic Island	Alkali basalt	3
T-12-07	Taumako	Oceanic Island	Alkali basalt	3
T-12-08	Taumako	Oceanic Island	Trachybasalt	3
T-12-09	Taumako	Oceanic Island	Alkali basalt	3
T-12-10	Taumako	Oceanic Island	Alkali basalt	3
K-12-24	Kapingamarangi	Oceanic Island	Picrite	4
K-12-25	Kapingamarangi	Oceanic Island	Picrobasalt	5
K-12-26	Kapingamarangi	Oceanic Island	Basanite	6
K-12-28	Kapingamarangi	Island Arc	Low-K basaltic andesite	7
K-12-29	Kapingamarangi	Island Arc	High-K calc-alkaline basalt	8

SI 4. Methodology of source assignments

Naturalistic approach to source determination

Our approach to provenance analysis draws on patterns of chemical heterogeneity in geological environments to demonstrate the movements of stone materials and artefacts in the archaeological record. In order to unambiguously assign artefacts to sources, we studied the trace element compositions and isotope chemistry to investigate how geochemical "fingerprints" of artefacts are compatible with specific regions. The Pacific islands are divided in two main categories: arc islands formed along the western Pacific subduction zones, and oceanic islands resulting from intraplate volcanism. Because they relate to different mantle sources and different melting processes, island arc basalts (IAB) and oceanic island basalts (OIB) present different geochemical characteristics, which can be used to broadly assess the geological origin of a sample, and provide a useful constrain when comparing artefacts and sources. Trace element and isotopic compositions of volcanic rocks relate to complex petrogenetic processes and vary in space and time depending on mantle source distributions. These can therefore be used to characterize raw material sources and archaeological items and reliably assess their geological provenance. Our naturalistic approach to geochemical sourcing enables us to consider a wider range of possible islands of origin for each artefact, including those where archaeological quarries are not yet known.

Methodology of source identification

Reference material and source data used to assess the provenance of the artefacts are accessed from two online open-access databases: GEOROC (https://georoc.eu/), a global geochemical database containing published chemical and isotopic data on geological materials (*44*), and Pofatu (https://pofatu.clld.org/), a database of geochemical compositions and contextual information for archaeological sources and artefacts (*38*). In order to identify the geological origin of each artefact, we compared the geochemical signatures with matching samples in both databases using SQL queries. These data requests expressed in Structured Query Language make it possible to extract subsets of samples based on specific variables such as targeted geochemical parameters, particular archipelagos and islands, or particular tectonic settings (convergent margins or oceanic islands) within the Asia-Pacific region. In order to find matching geochemical compositions in both databases, geochemical parameters were assigned range of values which were set according to the

natural variability of each chemical parameter and the analytical precision of present and past measuring instruments (Table S7). Specific SQL queries were then used to extract matching source data from GEOROC and Pofatu databases, according to their isotopic and geochemical compositions. For analyses using isotopic compositions, source data was extracted based on all 5 isotopic ratios; for analyses using major and trace elements, source data was extracted based on SiO_2 and K_2O contents when looking for IAB, and based on SiO_2, Na_2O and K_2O contents, as well as Pb isotopic data (Table S8). Matching source data were then compared with our Polynesian Outlier artefacts.

Table S7. Range values for SQL queries. Table credit: Aymeric Hermann.

**Major elements**	**Trace elements**	**Isotopes (IAB)**	**Isotopes (OIB)**
SiO_2 ± 1.5 p.p.	Cs, Rb, Ba, Th ± 50%	$^{143}Nd/^{144}Nd$ ± 0.1‰	$^{143}Nd/^{144}Nd$ ± 0.1‰
MgO ± 1.5 p.p.	Ce, Pb, Nd, Sr ± 50%	$^{87}Sr/^{86}Sr$ ± 0.1‰	$^{87}Sr/^{86}Sr$ ± 0.1‰
Na_2O ± 1.5 p.p.	Nb, Sm, Zr, Yb ± 50%	$^{206}Pb/^{204}Pb$ ± 0.5%	$^{206}Pb/^{204}Pb$ ± 1%
K_2O ± 1.5 p.p.		$^{207}Pb/^{204}Pb$ ± 0.5%	$^{207}Pb/^{204}Pb$ ± 1%
		$^{208}Pb/^{204}Pb$ ± 0.5%	$^{208}Pb/^{204}Pb$ ± 1%

SI 5. Data Visualization

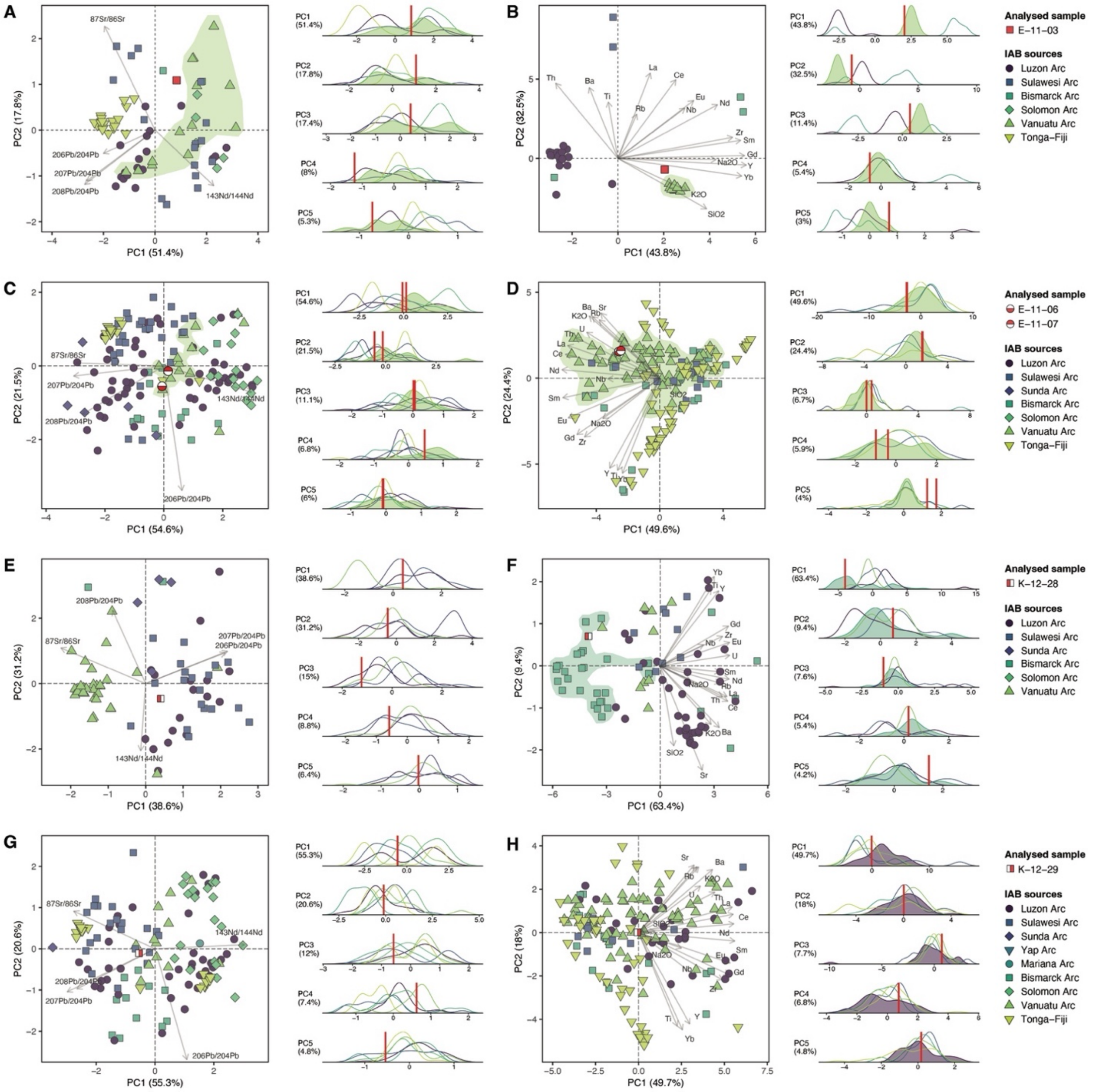

Figure S10. Assignment of IAB-related artefacts to source archipelagos. Each panel (**A** to **H**) show Principal Component Analysis plots (PCA) and associated density plots of the first 5 principal components with samples marked with a red segment. Plots of isotopic compositions are shown in left panels and of elemental compositions in right panels. The proportion of explained variance is indicated in parentheses. Figure credit: Aymeric Hermann.

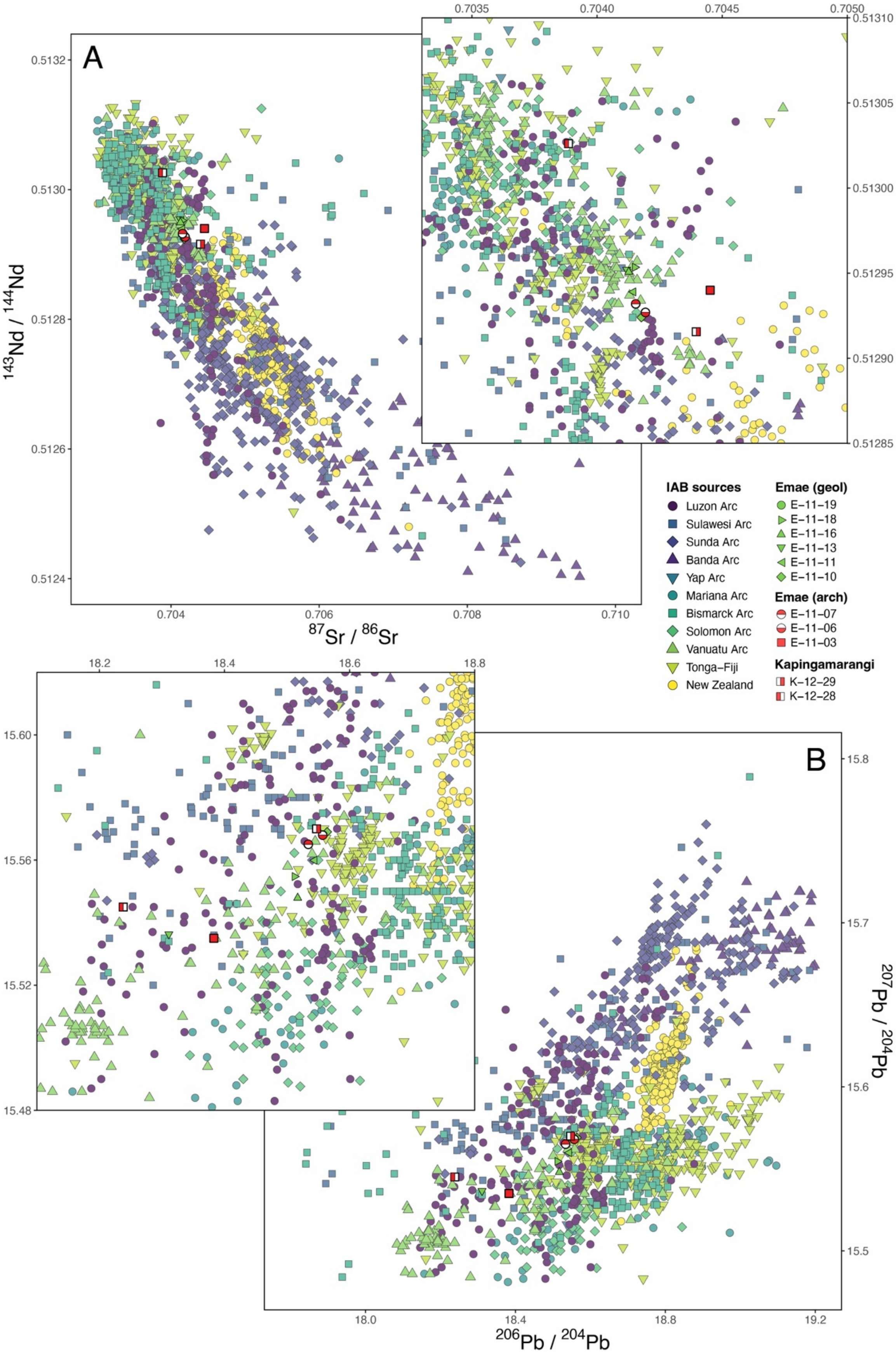

Figure S11. Plots of Nd, Sr and Pb isotopic compositions for Island Arcs and IAB-related artefacts. Figure credit: Aymeric Hermann.

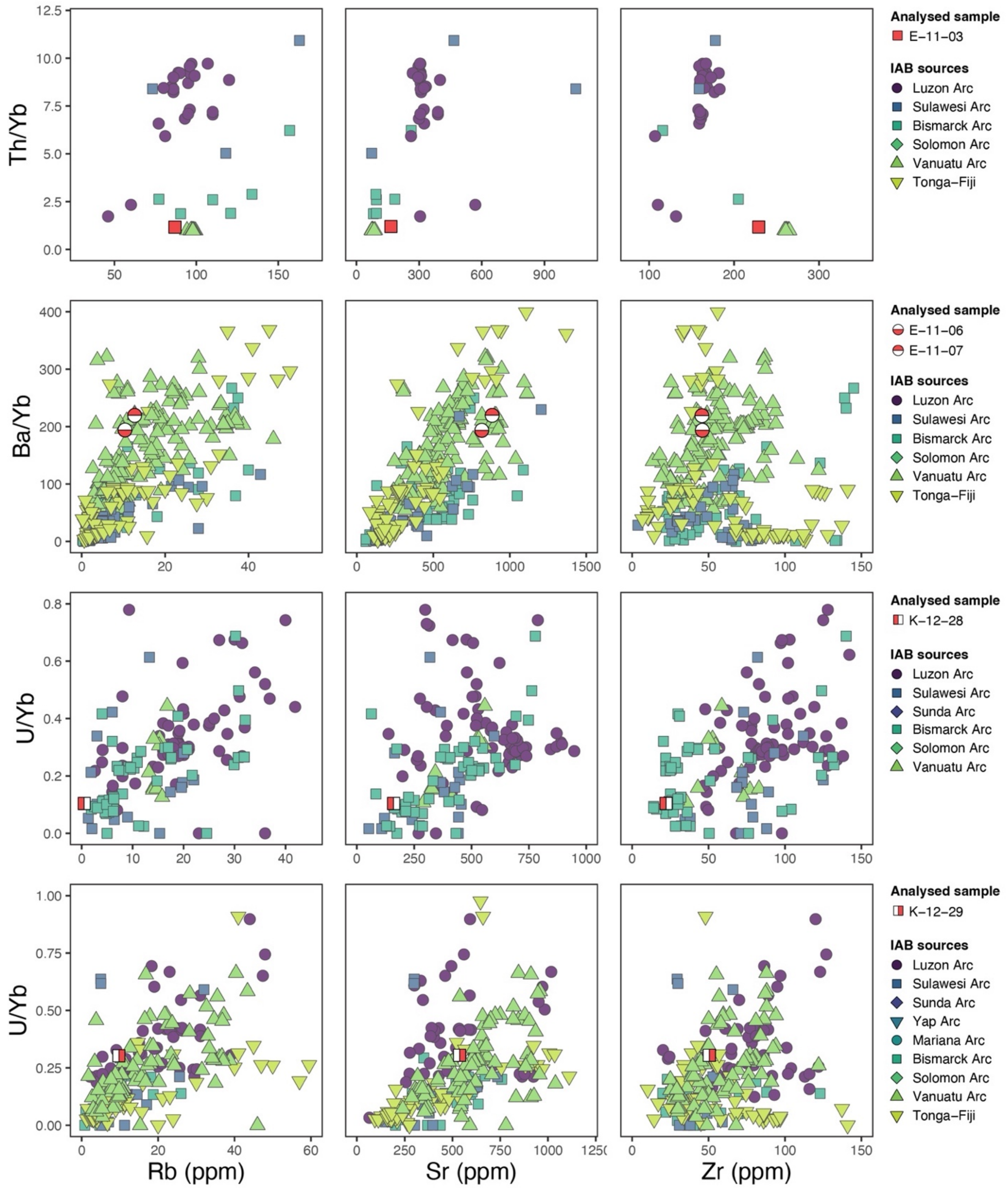

Figure S12. Biplots of selected trace elements for Island Arcs and IAB-related artefacts. Figure credit: Aymeric Hermann.

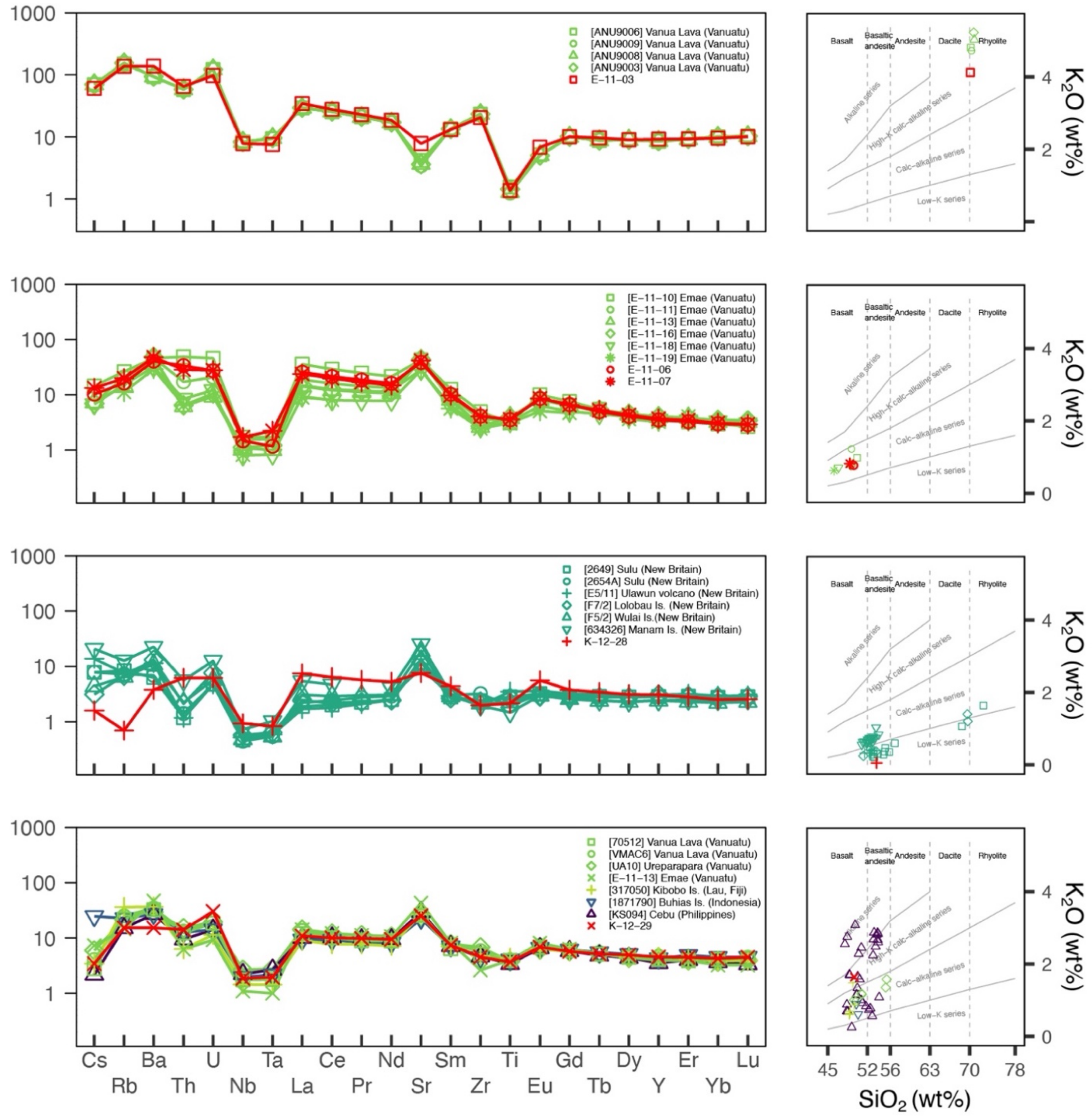

Figure S13. Multi-element plots normalized to the Primitive mantle (*98*) **and K_2O versus SiO_2 plots** (*99*) **for Island Arcs and IAB-related artefacts.** Figure credit: Aymeric Hermann.

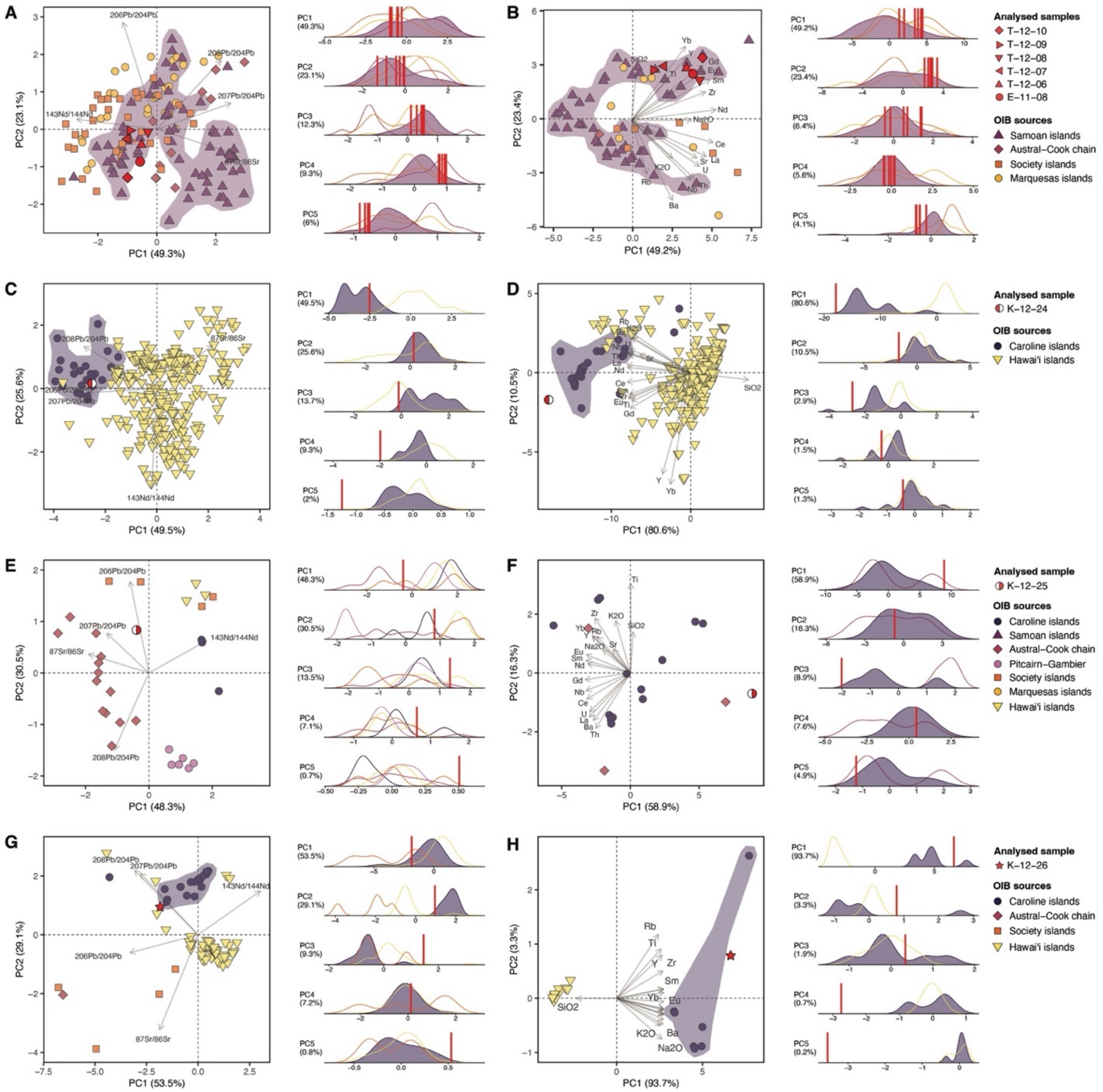

Figure S14. Assignment of OIB-related artefacts to source archipelagos. Each panel (**A** to **H**) show Principal Component Analysis plots (PCA) and associated density plots of the first 5 principal components with samples marked with a red segment. Plots of isotopic compositions are shown in left panels and of elemental compositions in right panels. The proportion of explained variance is indicated in parentheses. Figure credit: Aymeric Hermann.

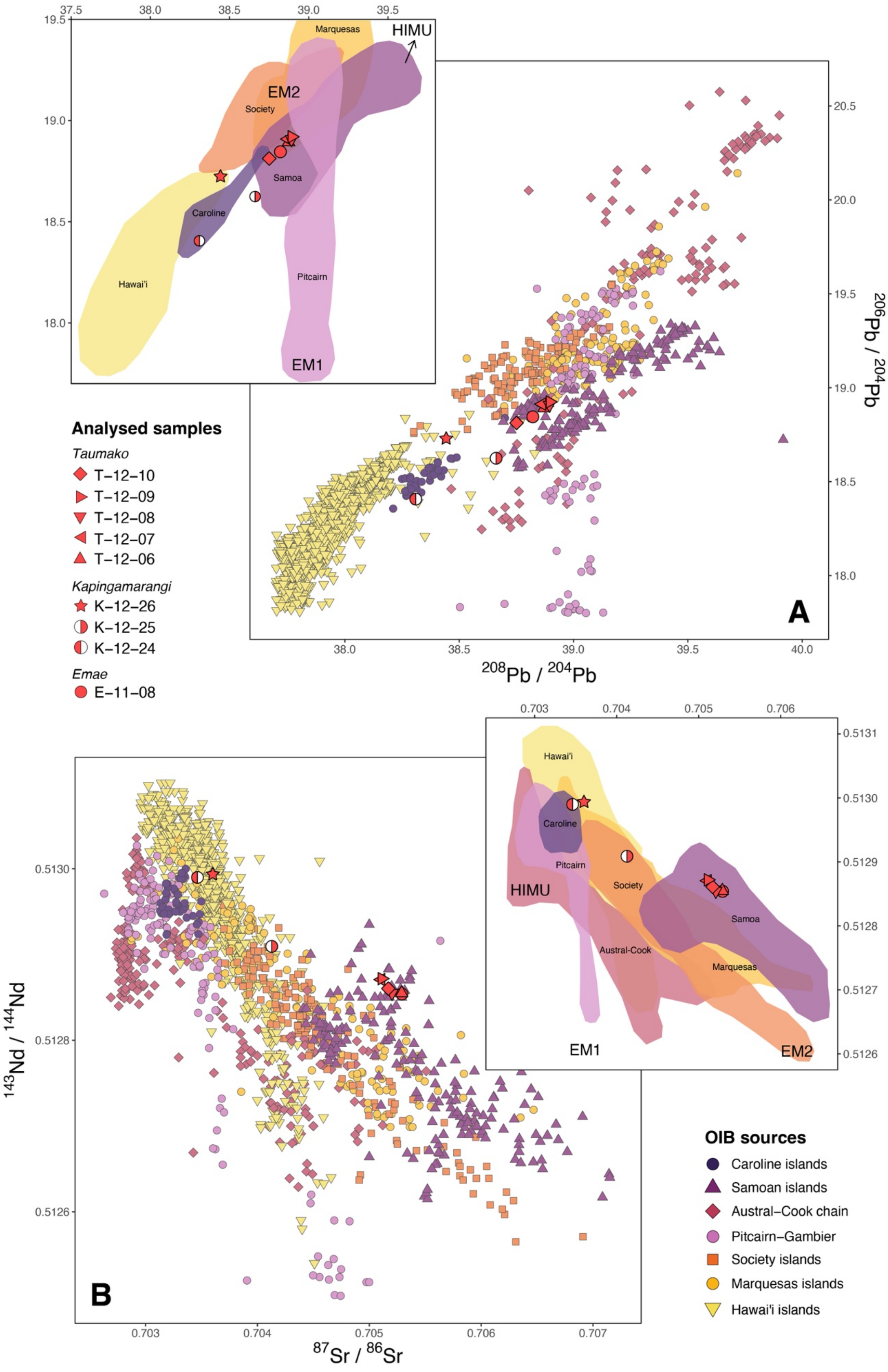

Figure S15. Plots of Nd, Sr and Pb isotopic compositions for Ocean Islands and OIB-related artefacts. Figure credit: Aymeric Hermann.

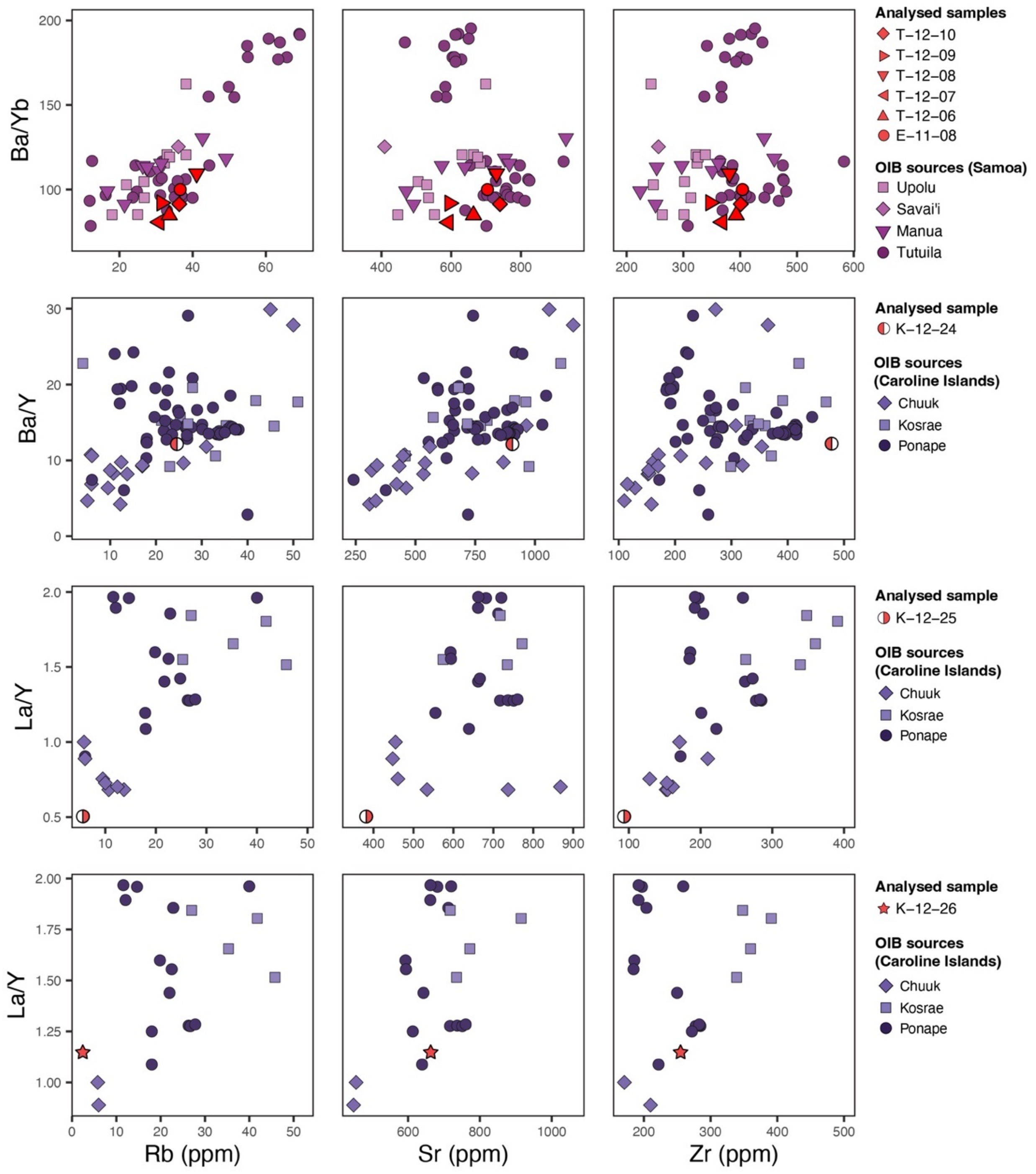

Figure S16. Biplots of selected trace elements for Ocean Islands and OIB-related artefacts. Figure credit: Aymeric Hermann.

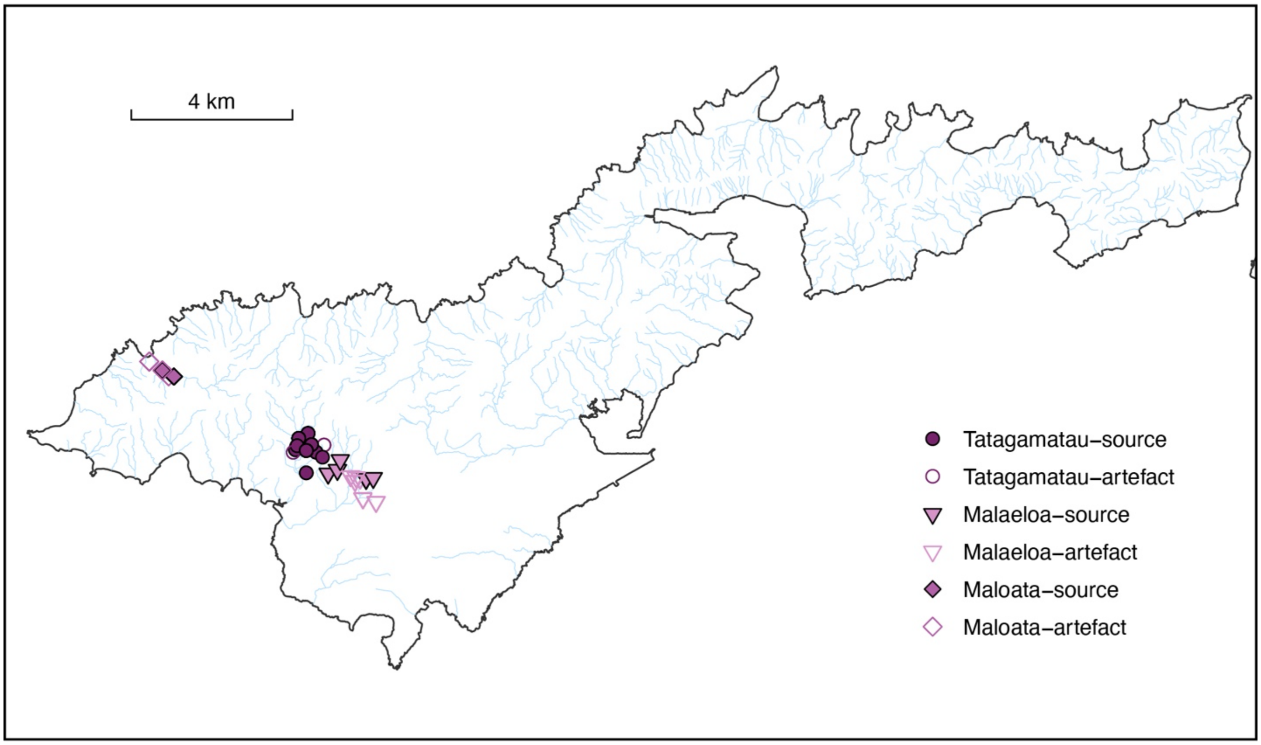

Figure S17. Location of west Tutuila archaeological sources. Figure credit: Aymeric Hermann.

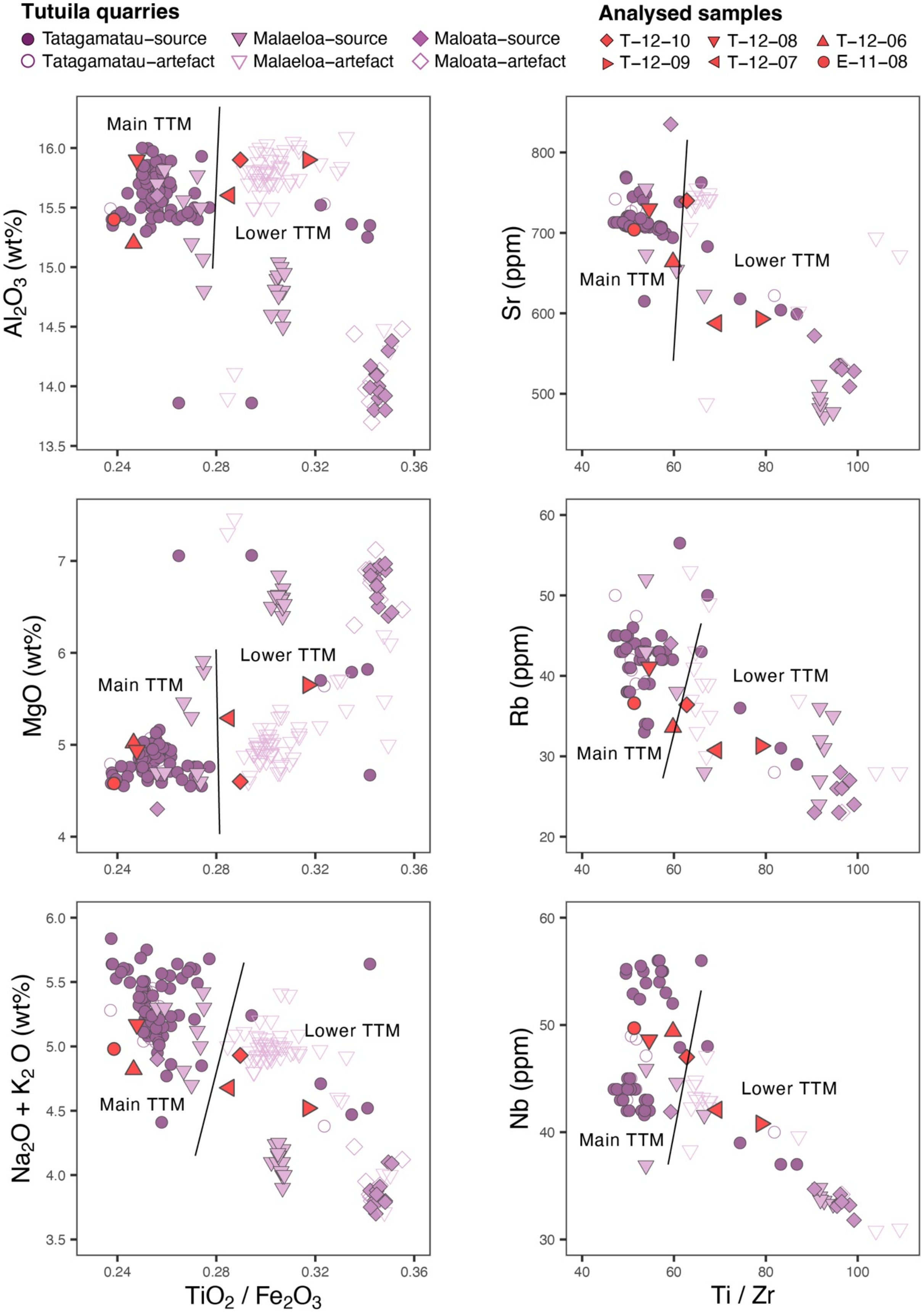

Figure S18. Biplots of selected oxides and trace elements for west Tutuila archaeological sources and related artefacts (this study). Figure credit: Aymeric Hermann.

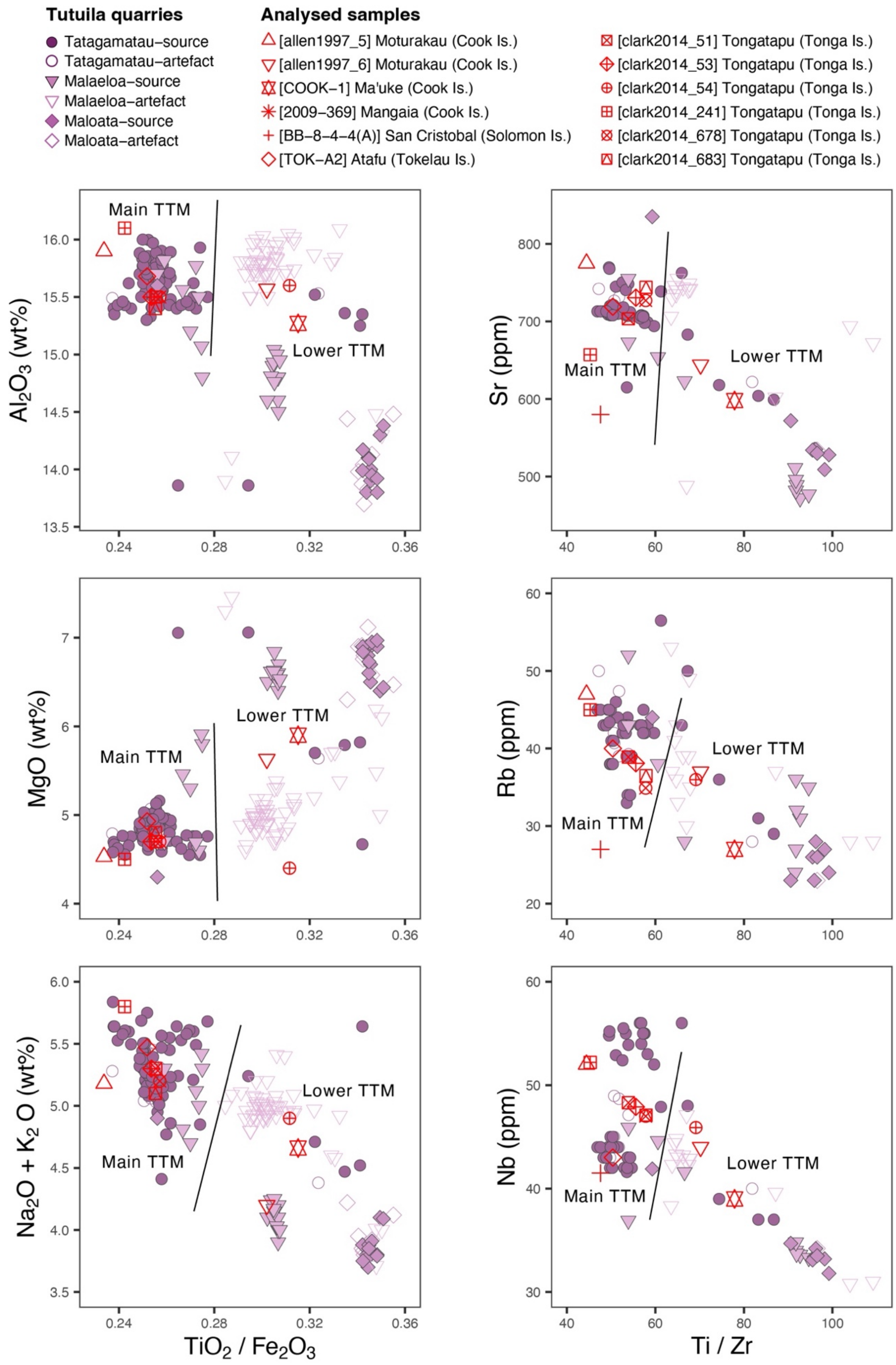

Figure S19. Biplots of selected oxides and trace elements for west Tutuila archaeological sources and related artefacts in the literature (*28, 33, 58, 61*). Figure credit: Aymeric Hermann.

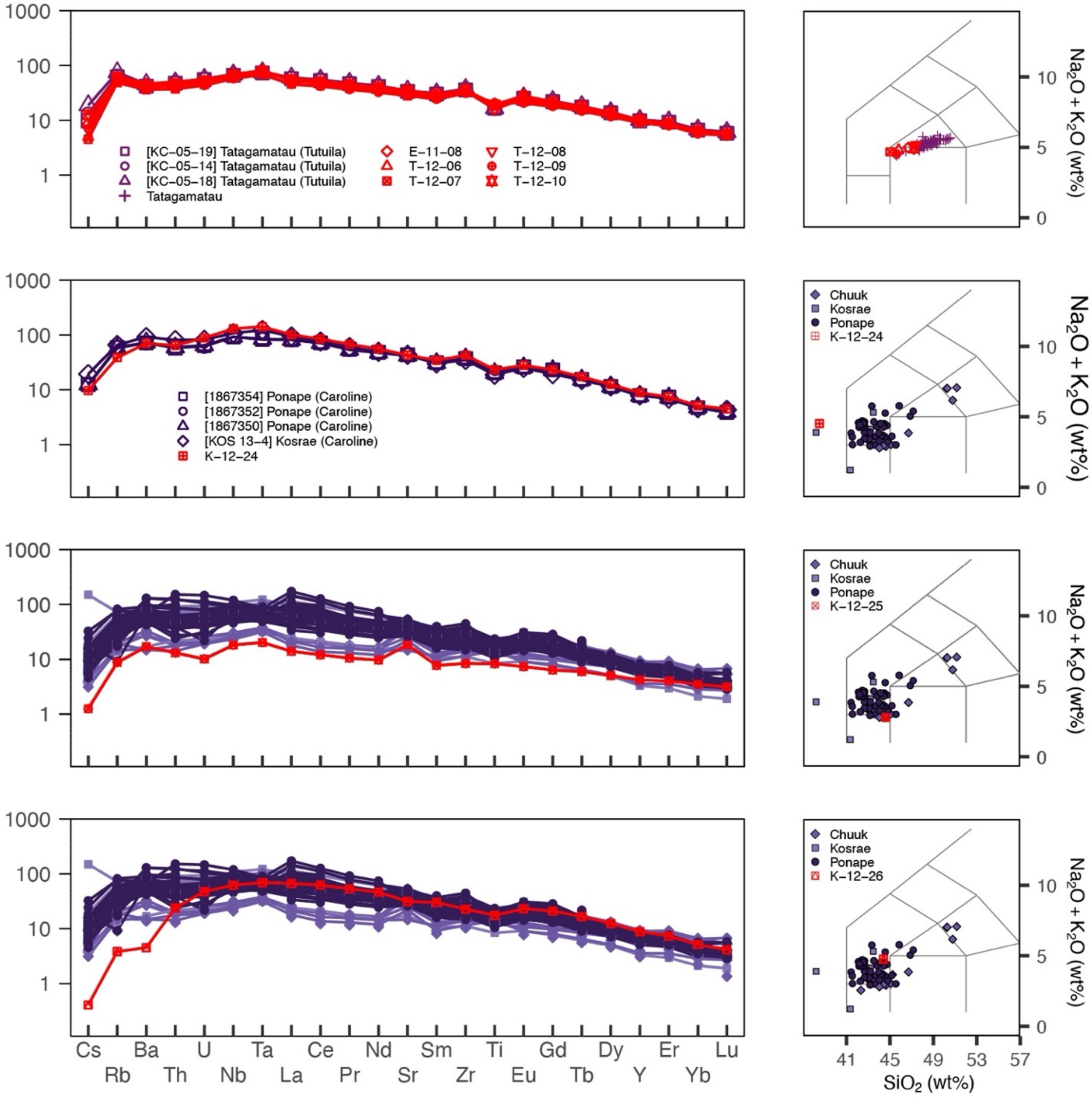

Figure S20. Multi-element plots normalized to the Primitive mantle (*98*) and Total Alkali Silica plots (*100*) for Ocean Islands and OIB-related artefacts. Figure credit: Aymeric Hermann.
